# Cochaperonin CPN20 negatively regulates abscisic acid signaling in *Arabidopsis*

**DOI:** 10.1007/s11103-013-0082-8

**Published:** 2013-06-20

**Authors:** Xiao-Feng Zhang, Tao Jiang, Zhen Wu, Shu-Yuan Du, Yong-Tao Yu, Shang-Chuan Jiang, Kai Lu, Xiu-Jing Feng, Xiao-Fang Wang, Da-Peng Zhang

**Affiliations:** MOE Systems Biology and Bioinformatics Laboratory, School of Life Sciences, Tsinghua University, Beijing, 100084 China

**Keywords:** Abscisic acid signaling, *Arabidopsis thaliana*, Cochaperonin CPN20, Mg-chelatase H subunit, WRKY40 transcription factor

## Abstract

Previous study showed that the magnesium-protoporphyrin IX chelatase H subunit (CHLH/ABAR) positively regulates abscisic acid (ABA) signaling. Here, we investigated the functions of a CHLH/ABAR interaction protein, the chloroplast co-chaperonin 20 (CPN20) in ABA signaling in *Arabidopsis thaliana*. We showed that down-expression of the *CPN20* gene increases, but overexpression of the *CPN20* gene reduces, ABA sensitivity in the major ABA responses including ABA-induced seed germination inhibition, postgermination growth arrest, promotion of stomatal closure and inhibition of stomatal opening. Genetic evidence supports that CPN20 functions downstream or at the same node of CHLH/ABAR, but upstream of the WRKY40 transcription factor. The other CPN20 interaction partners CPN10 and CPN60 are not involved in ABA signaling. Our findings show that CPN20 functions negatively in the ABAR-WRKY40 coupled ABA signaling independently of its co-chaperonin role, and provide a new insight into the role of co-chaperones in the regulation of plant responses to environmental cues.

**Electronic supplementary material:**

The online version of this article (doi:10.1007/s11103-013-0082-8) contains supplementary material, which is available to authorized users.

## Introduction

Phytohormone abscisic acid (ABA) regulates many developmental processes including embryo maturation, seed germination and seedling growth, and is a key hormone in plant adaptation to adverse conditions such as drought, salt and cold stresses (reviewed in Finkelstein and Rock 2002; Adie et al. 2007; Cutler et al. 2010). ABA signal transduction has been extensively studied, and numerous signaling components, including ABA receptors, have been identified (reviewed in Finkelstein and Rock 2002; Cutler et al. 2010). A putative G-protein-coupled receptor GCR2 and GPCR-type G proteins GTG1 and GTG2 have been reported as candidate plasma membrane-type ABA receptors (Liu et al. 2007a, b; Johnston et al. 2007; Pandey et al. 2009), though it is controversial whether GCR2 regulates ABA-mediated inhibition of seed germination and post-germination growth (Gao et al. 2007; Guo et al. 2008). GTGs are positively involved in ABA signaling and interacts with the sole *Arabidopsis* G protein α subunit GPA1 (Pandey and Assmann 2004), which inhibits the activity of GTG-ABA binding to negatively regulate ABA signaling (Pandey et al. 2009). The members of the START-domain superfamily proteins PYR/PYL/RCAR have been identified as cytosolic ABA receptors, which mediate a core ABA signaling pathway (Ma et al. 2009; Park et al. 2009; Santiago et al. 2009; Cutler et al. 2010). The type 2C protein phosphatases (PP2Cs), functioning directly downstream of the PYR/PYL/RCAR receptors, inhibit SNF1-related protein kinase 2s (SnRK2s), which phosphorylate an ABF/AREB/ABI5 clade of bZIP-domain transcription factors to induce ABA responses (Ma et al. 2009; Park et al. 2009; Santiago et al. 2009; Cutler et al. 2010).

We previously reported that the chloroplast magnesium-protoporphyrin IX chelatase large subunit (Mg-chelatase H subunit CHLH/putative ABA receptor ABAR) functions as a candidate receptor for ABA in *Arabidopsis thaliana* (Shen et al. 2006; Wu et al. 2009; Du et al. 2012), which antagonizes a group of WRKY-domain transcription repressors to relieve ABA-responsive genes of inhibition (Shang et al. 2010; Liu et al. 2012; Yan et al. 2012). Although it is controversial whether CHLH/ABAR binds ABA (Shen et al. 2006; Muller and Hansson 2009; Wu et al. 2009; Tsuzuki et al. 2011; Wang et al. 2011; Du et al. 2012), it has been well supported that CHLH/ABAR functions in ABA signaling (Shen et al. 2006; Wu et al. 2009; Shang et al. 2010; Du et al. 2012). There are four *abar* mutant alleles in *Arabidopsis*, *abar*-*2*, *abar*-*3*, *cch* and *rtl1*, which all show altered ABA responses (Shen et al. 2006; Wu et al. 2009; Tsuzuki et al. 2011; Du et al. 2012). Evidence from independent groups revealed that CHLH/ABAR mediates ABA signaling in guard cells in both *Arabidopsis* (Legnaioli et al. 2009; Tsuzuki et al. 2011) and peach (*Prunus persica*) leaves (Jia et al. 2011a). We recently showed that CHLH/ABAR also regulates guard cell signaling in response to ABA in tobacco (*Nicotiana benthamiana*) leaves (Du et al. 2012). Additionally, it has been demonstrated that CHLH/ABAR mediates ABA signaling in fruit ripening of both peach (Jia et al. 2011a) and strawberry (*Fragaria ananassa*) (Jia et al. 2011b). These data demonstrate that CHLH/ABAR is an essential ABA signaling regulator in plant cells. However, the mechanism of the ABAR/CHLH-mediated signaling pathway remains largely unclear, and screening of key components functioning in this pathway is necessary for understanding the highly complex mechanisms of ABA signal transduction.

Chaperones are a group of functional accompanying proteins, which play a central role in defining the balance of protein folding, assembly and degradation in both optimal and adverse conditions (Wang et al. 2004). Expression of most chaperones can be up-regulated by heat or other stresses, and hence they are usually referred to as heat-shock proteins (HSPs). To date, five major families of chaperones have been described in plants according to their molecular weight: the HSP100 family, the HSP90 family, the HSP70 family, the chaperonins (HSP60), and the small HSP family (Weiss et al. 2009). Co-chaperones are proteins that interact with chaperones such as HSP60, HSP70 or HSP90 to assist in folding specific substrates. Recently, they have been reported to take part in some signaling pathways together with their corresponding chaperones. HSP40, for instance, functions as a co-chaperone and stimulates the ATPase activity of HSP70, playing an important role in the adaptation process in response to increased salt concentration in plants (Zhao et al. 2010). It has been reported that the HSP40-like protein J3 (bacterial DnaJ homolog 3) is involved in the PKS5 (Salt Overly Sensitive2-Like Protein Kinase5)-mediated signaling pathway in salt tolerance in *Arabidopsis*, where J3 could activate plasma membrane H^+^-ATPase activity by repressing PKS5 kinase activity (Yang et al. 2010). SGT1, acting as a scaffold to bridge HSP70/HSP90 complexes (Catlett and Kaplan 2006), is also important to auxin and jasmonic acid (JA)-mediated signaling (Gray et al. 2003) and SCF E3 ubiquitin ligase-dependent signaling in plants (Kitagawa et al. 1999). Most recently, HSP90 and SGT1b were identified as negative regulators in ABA-induced seed germination inhibition and stomatal closure (Clément et al. 2011).

Among the best studied chaperones/co-chaperones are chaperonins HSP60 or CPN60/co-chaperonin CPN10. Chaperonin HSP60/CPN60 include two groups. Group I chaperonins are found in bacteria, eukaryotic chloroplasts and mitochondria. In *Escherichia* *coli* (*E*. *coli*), the barrel-like chaperonin GroEL/CPN60 contains a hydrophobic apical domain, which, with the help of co-chaperonin GroES/CPN10, could form a hydrophilic cage inside where encapsulated proteins fold freely in a short time. The folded proteins escape from the cage after the GroES/CPN10 dissociation from GroEL/CPN60 (Hartl et al. 2011). Group II chaperonins are found in archaea and in the cytosol of eukaryotic cells as well, which contain a built-in lid domain functioning similarly as GroES/CPN10 (Hartl et al. 2011).

Chloroplast CPN20, a co-chaperonin of CPN60, was first isolated from pea chloroplast lysate (Hill and Hemmingsen 2001). It consists of a transit peptide and two homologous CPN10-like domains, which exhibit 46 % amino acid sequence identity to each other and are joined together head-to-tail by a short link region (Beneyx et al. 1995; Hirohashi et al. 1999; Koumoto et al. 1999; Sharkia et al. 2003; Weiss et al. 2009). Analysis of chaperonin gene expression in *Arabidopsis* showed that CPN20 is strongly expressed on its own, with only a weak appearance of potential partners in the CPN60 family (Weiss et al. 2009), and that CPN20 seems to be the most highly expressed co-chaperonin protein among all of the three co-chaperonin proteins (two CPN10s, and one CPN20) in chloroplasts (Peltier et al. 2006). This indicates that CPN20 may have some other functions independent of its co-chaperonin role (Weiss et al. 2009). Indeed, a most recent report demonstrated that CPN20 mediates iron superoxide dismutase (FeSOD) activity independent of its co-chaperonin role in the *Arabidopsis* chloroplasts (Kuo et al. 2013).

Here, we report that CPN20 is an interaction partner of the ABAR/CHLH protein, and is negatively involved in ABA signaling independently of its co-chaperonin role, and that CPN20 functions downstream or at the same node of ABAR and upstream of the WRKY40 transcription factor, which links CPN20 to the ABAR-WRKY40 coupled signaling pathway. These findings help to understand mechanisms of ABA signaling pathways, and provide a new insight into the role of co-chaperones in the regulation of plant responses to environmental cues.

## Materials and methods

### Plant materials and growth conditions

*Arabidopsis thaliana* ecotype Col-0 was used as the wild-type control. The T-DNA insertion lines in the *CPN10(1)*, *CPN10(2)* or *CPN20* gene were all obtained from the Arabidopsis Biological Resource Center (ABRC). The mutant lines were genotyped by PCR and DNA gel-blot analysis, and the exact position was determined by sequencing. Primers used for screening and genotyping were presented in Table S1. The Col-0, *wrky40*-*1* and *cpn20*-*1* mutants were used to create *CPN20*-overexpressing transgenic lines and complementation lines. The open reading frame (ORF) of *CPN20* was amplified by PCR from Col-0 cDNA using the primers presented in Table S1 and cloned into the pCAMBIA-1300-221 vector that contains the cauliflower mosaic virus 35S promoter. The construct, verified by sequencing, was introduced into the GV3101 strain of *Agrobacterium tumefaciens*. The construct was used to transform plants of wild-type Col-0 or *wrky40*-*1* mutant by floral infiltration. Transgenic plants were selected by hygromycin resistance and confirmed by PCR. The homozygous T3 seeds were used for further analysis. The *ABAR*-RNAi construct described previously (Shen et al. 2006) was used to transform directly into the *cpn20*-*1* mutant. The ABAR levels were analyzed by real-time PCR and immunoblotting, and decreased levels of *ABAR* mRNA and protein products were detected in different RNAi lines. The homologous T3 generation seeds or plants were used for analysis. The *cch* mutant (with ecotype Columbia as background) was a generous gift from Dr. J. Chory (The Salk Institute, La Jolla, CA). Double mutants were generated by genetic crossing and identified by PCR genotyping. Accession numbers of all the genes and mutants used in this study are listed in Supporting Information. *Arabidopsis* seeds were disinfected and plated on MS medium (Sigma) supplemented with 3 % sucrose and 0.8 % agar (pH 5.9), chilled for 3 days at 4 °C and transferred to a growth chamber at ~80 μmol photons m^−2^ s^−1^ or in compost soil at ~120 μmol photons m^−2^ s^−1^ using cool white fluorescent lamps under a 16 h-light/8 h-dark photoperiod and 60 % relative humidity.

### Yeast two-hybrid assay

The Matchmaker Gal4 two-hybrid system (Clontech) was used for yeast two-hybrid screening of the *Arabidopsis* cDNA library from ABRC. The ORF of the middle fragment (encoding amino acid residues 348–1038) of *ABAR* was fused to GAL4 DNA binding domain in the plasmid pGBKT7. Yeast transformants were thoroughly selected on synthetic dropout medium (SD medium deficient in the nutrients Leu, Trp, His, and Ade) based on the manufacturer’s instructions (Clontech). For analysis of protein interaction by two-hybrid assay, the full-length or partial sequences of *CPN20* were inserted into prey plasmid pGADT7 and the truncated *ABAR*s were cloned into bait plasmid pGBKT7. Primers used in the vector construction are presented in Table S1. Different combinations of plasmids were transformed into the yeast strain AH109. Transformants were plated on Leu-Trp-deficient and Leu-Trp-His-Ade-deficient medium respectively and grew for 5–7 days at 30 °C. The empty vectors pGBKT7 and pGADT7 were used as a negative control, while pGBKT-53 and pGADT7-T were used as a positive control. Expression of different fusion proteins was detected by immunoblot experiments. The ABARs-BD were analyzed using anti-Myc antibody and the CPN20s-AD were analyzed using anti-HA antibody. The β-galactosidase assay is described in Supporting Information.

### Other techniques

Production of anti-CPN20 serum, real-time PCR analysis, CoIP assays in yeast and in planta, luciferase complementation imaging (LCI), and phenotypic analysis are described in Supporting Information.

## Results

### CPN20 is an interaction partner of ABAR

**Fig. 1:**
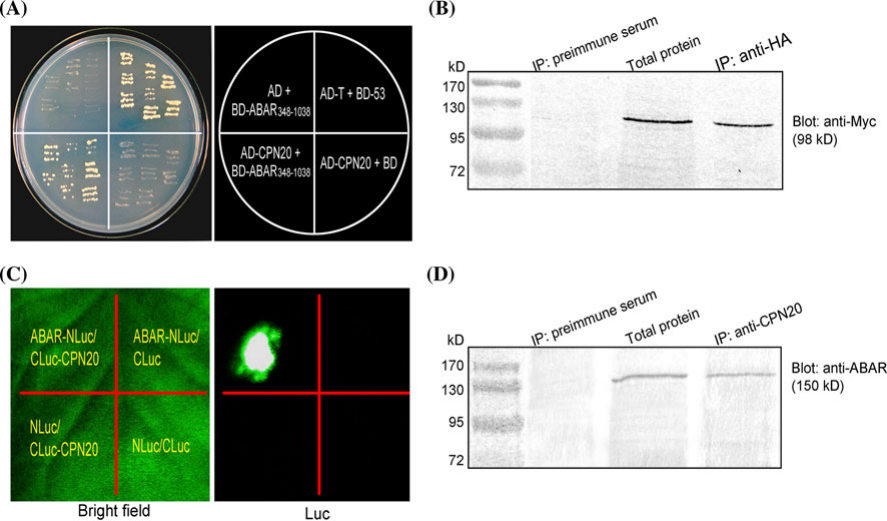
CPN20 interacts with ABAR. **a** Validation of the interaction between CPN20 and ABAR in the yeast cells grown on drop-out selection medium lacking Trp, Leu, His, and Ade (SD4−). The open reading frame (ORF) of the middle fragment (amino acid residues [A.A.] 348–1038) of *ABAR* was fused with the DNA binding domain in the bait vector pGBKT7 (BD-ABAR_348–1038_), while the full-length sequence of *CPN20* was fused with activation domain in the prey vector pGADT7 (AD-CPN20). Yeast strain AH109 co-transformed with the AD-CPN20/BD-ABAR_348–1038_ constructs was able to grow on SD4−. BD-53/AD-T was taken as a positive control. AD/BD-ABAR_348–1038_ and AD-CPN20/BD were taken as negative controls. **b** Co-immunoprecipitation (Co-IP) assays in yeast cells. ABAR and CPN20 were co-immuno-precipitated from yeast total proteins. IP, immunoprecipitation; Blot, immunoblot; anti-HA and anti-c-Myc, antiserum specifically against HA (linked to CPN20) and c-Myc (linked to ABAR) epitope tag, respectively. Immunoprecipitation with preimmune serum was taken as a negative control. **c** Validation of the interaction between CPN20 and ABAR by firefly luciferase complementation imaging (LCI) assay. ABAR was fused with N-terminal fragment of LUC (NLuc) to generate ABAR-NLuc. CPN20 was fused with the C-terminal fragment of LUC (CLuc) to generate CLuc-CPN20. Tobacco leaves were transformed with the construct pairs as indicated in the left panel. The left panel shows the bright field and the right panel the corresponding luciferin fluorescence of a treated leaf. **d** Co-immunoprecipitation (Co-IP) assays in plants. ABAR and CPN20 were co-immunoprecipitated from *Arabidopsis* total proteins. IP, immunoprecipitation; Blot, immunoblot; anti-CPN20 and anti-ABAR, antiserum specifically against CPN20 and ABAR, respectively. Immunoprecipitation with preimmune serum was taken as a negative control. The experiments in **a** to **d** were repeated three times with the same results

We used a fragment encoding the middle region of ABAR [amino acid residues (A.A.) 348–1038, abbreviated as ABAR_348–1038_] as a bait to screen the *Arabidopsis* cDNA library by yeast two-hybrid system. Sequence analysis of all the presumed clones showed that a chloroplast co-chaperonin CPN20 is one of the candidate interaction partners of ABAR. Further assays showed that the yeast cells co-expressing CPN20 and ABAR_348–1038_ or AD (activation domain)-T7 and BD (DNA binding domain)-53 (a positive control) were able to grow on synthetic drop-out selection medium lacking Leu, Trp, His, and Ade (SD-Leu-Trp-His-Ade, SD4−) (Fig. 1a). Empty vectors pGBKT7 harboring BD and pGADT7 harboring AD were used as negative controls, and showed no interaction with CPN20 or ABAR_348–1038_ (Fig. 1a), indicating that the detected interaction between CPN20 and ABAR in the yeast system is specific. We confirmed this physical interaction by co-immunoprecipitation (CoIP) assay in yeast (Fig. 1b).

The interaction between ABAR and CPN20 was further tested in vivo using both a firefly luciferase (LUC) complementation imaging (LCI) assay in tobacco (*N. benthamiana*) and CoIP in *Arabidopsis*. In the LCI assay, the N-terminus of LUC was ligated to the full length ABAR (ABAR-NLuc) and the C-terminus of LUC was fused with the full length CPN20 (CLuc-CPN20). Strong LUC activity was detected in leaves co-transformed with ABAR-NLuc and CLuc-CPN20 vectors, whereas no LUC activity was detected in the leaves co-infiltrated with the negative-control construct pairs ABAR-NLuc and CLuc, CLuc-CPN20 and NLuc, or NLuc and CLuc (Fig. 1c). In the CoIP assay in the *Arabidopsis* total protein extracts, the anti-CPN20 serum (Supplementary Fig. 1) (IP: anti-CPN20) pulled down ABAR protein (~150 kD), which was immuno-blotted with the anti-ABAR serum (Wu et al. 2009) (Blot: anti-ABAR), while the preimmunue serum (IP: preimmune serum), served as a negative control, did not pull down any significant immuno-signal (Fig. 1d). These data showed that CPN20 specifically interacts with ABAR in vivo.

### The *CPN20* gene is ubiquitously expressed and the CPN20 protein is localized to the chloroplast stroma

*CPN20* is a single-copy gene in *Arabidopsis* genome. Previous study reported that the *Arabidopsis* *CPN20* gene is expressed in almost all the tissues and all the developmental stages (Peltier et al. 2006; Weiss et al. 2009). We confirmed this expression profile with the *CPN20*-promoter-GUS (β-glucuronidase) transgenic lines, which showed that CPN20 is expressed ubiquitously in different tissues except for dry seeds (Supplementary Fig. 2a). These data are essentially consistent with the microarray data in the public website Genevestigator (http://www.genevestigator.com). It is notable that the *CPN20* gene is expressed at a higher level especially in germinated seed and seedlings compared to other two chloroplast co-chaperonin 10 (*CPN10*) genes (Supplementary Fig. 2b). Transient expressions of the CPN20-GFP fusion protein in *Arabidopsis* protoplasts showed that CPN20 resides mainly in chloroplast stroma space (Supplementary Fig. 3a, b). Further transient co-expression of the ABAR-GFP and CPN20-mCherry fusion proteins in *Arabidopsis* protoplasts showed that ABAR and CPN20 partly co-localize in stroma and particularly around the periphery of the chloroplast (Supplementary Fig. 3c), which provides opportunity for the interaction of CPN20 with ABAR in vivo.

### Down-expression of *CPN20* increases, but overexpression of *CPN20* reduces, ABA sensitivity in the major ABA responses

**Fig. 2:**
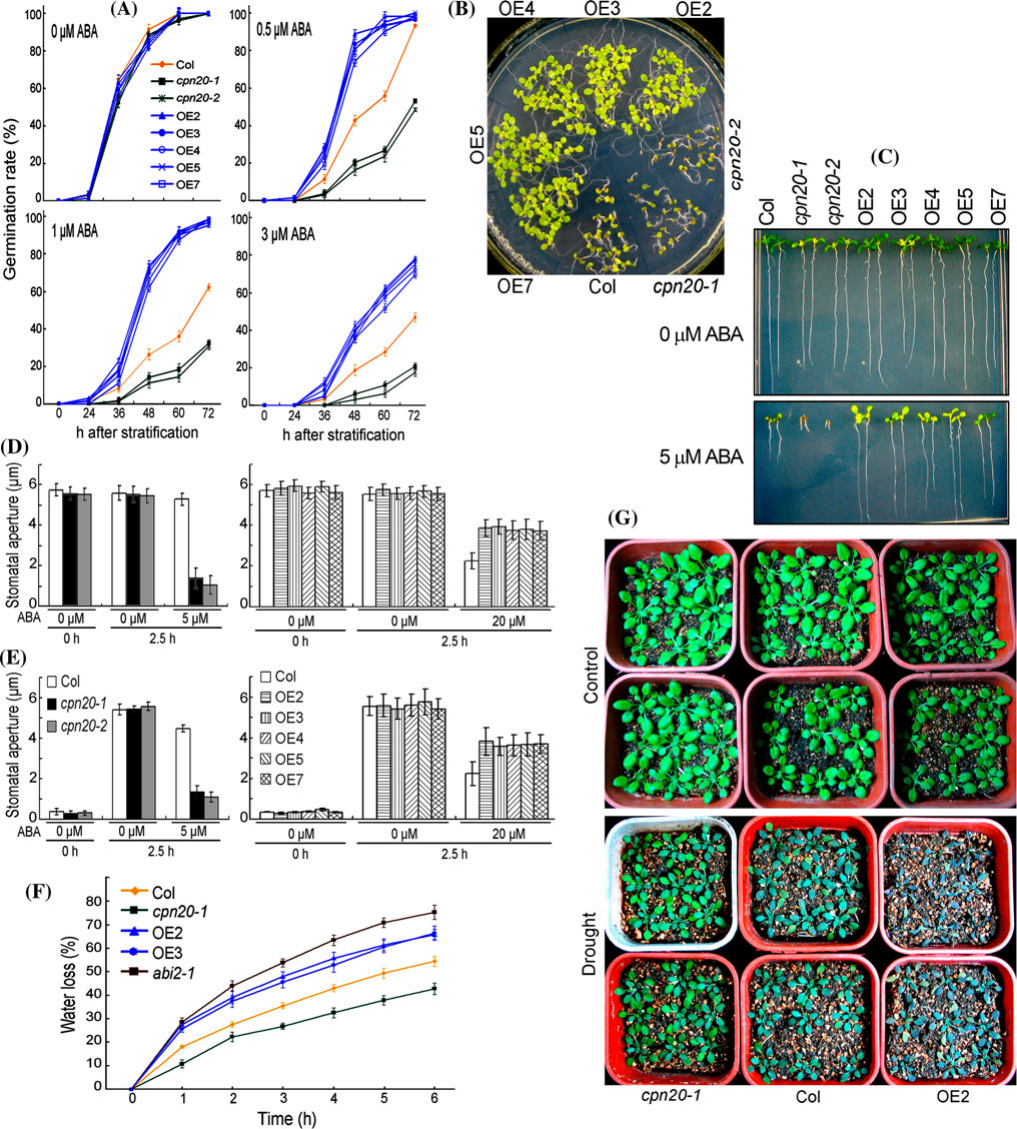
CPN20 negatively regulates ABA signaling. **a** Seed germination: germination rates of the wild-type Col, *cpn20*-*1* and *cpn20*-*2* mutants, and five *CPN20*-overexpressing lines (OE2, OE3, OE4, OE5 and OE7) were recorded on ABA-free (0 μM) and ABA-containing (0.5, 1 or 3 μM) medium from 24 to 72 h after stratification. Each value is the mean ± SE of five biological determinations. **b** Early seedling growth: seeds from the different genotypes as described in **a** were directly planted in MS medium supplemented with 0.5 μM (±)ABA, and the growth was investigated 12 days after stratification. **c** Early seedling growth: germinating seeds of the different genotypes as described in **a** were transferred, 48 h after stratification, from ABA-free medium to the medium supplemented with 0 or 5 μM (±)ABA, and the growth was investigated 10 days after the transfer. **d** and **e** ABA-induced stomatal closure (**d**) and ABA-induced inhibition of stomatal opening (**e**) of the different genotypes. Values are the mean ± SE from five independent experiments; n = 60 apertures per experiment. **f** Water loss rates during a 6-h period from the detached leaves of wild-type Col, *cpn20*-*1*, two CPN20-overexpressing lines (OE2 and OE3) and *abi2*-*1* mutant. Each value is the mean ± SE of five biological determinations. **g** Whole-plant status in the water loss assays for wild-type Col, *cpn20*-*1* mutant and a CPN20-overexpressiong line (OE2). Plants were well watered (Control) or drought stressed by withholding water (Drought) for 15 days. The experiments were replicated three times with similar results

We obtained three T-DNA insertion mutants in *CPN20* gene (Supplementary Fig. 4) (Col ecotype background) from Arabidopsis Biological Resource Center (ABRC), which were named *cpn20*-*1*, *cpn20*-*2* and *cpn20*-*3*. However, homozygous *cpn20*-*3* mutant is lethal. Real-time PCR and immunoblotting assay demonstrated that the expression level of *CPN20* is reduced in both *cpn20*-*1* and *cpn20*-*2* mutants (Supplementary Fig. 4a, b). Also, we generated *CPN20*-overexpressing lines (Supplementary Fig. 4c). We observed that the seeds of both *cpn20*-*1* and *cpn20*-*2* mutants showed ABA-hypersensitive phenotype, while the seeds of the five representative over-expression lines displayed ABA insensitive phenotype, in ABA-inhibited seed germination (Fig. 2b).

We adopted two approaches to assess the effects of CPN20 on the response of seedling growth to ABA: seeds were directly planted in (±)ABA-containing MS medium (Fig. 2b), or germinating seeds were transferred 48 h after stratification from the ABA-free medium to (±)ABA-containing medium (Fig. 2c). Similar results were obtained with these two approaches. Seedling growth of both *cpn20*-*1* and *cpn20*-*2* mutants showed ABA-hypersensitive phenotype, but the seedling growth of the over-expression lines displayed ABA insensitive phenotype (Fig. 2b, c; Supplementary Fig. 5a).

As regards stomatal response to ABA, we observed that both of the *cpn20* mutants exhibited ABA-hypersensitive phenotypes, while all the over-expression lines showed ABA-insensitive phenotypes in ABA-induced promotion of stomatal closure and inhibition of stomatal opening (Fig. 2d, e). Consistently, under dehydration conditions, the detached leaves of the *cpn20*-*1* mutant lost less water, but the detached leaves of the OE2 and OE3 lines lost more water than those of the wild type (Fig. 2f; Supplementary Fig. 6). The *abi2*-*1* dominant mutant (Leung et al. 1997) was used as a control, of which the detached leaves lost water more rapidly than those of the OE2 and OE3 lines (Fig. 2f; Supplementary Fig. 6). Also, we observed that the *cpn20*-*1* mutant showed higher capacity to conserve its water at the whole-plant level but the OE2 line showed less resistant to drought than the wild-type plants (Fig. 2g).

We introduced the *CPN20* cDNA into the *cpn20*-*1* mutant by generating transgenic lines, and obtained four lines with the *CPN20* levels similar to or slightly higher than those of wild-type plants. These *CPN20*-transgenic lines of the *cpn20*-*1* mutant showed essentially wild-type ABA sensitivity or slightly ABA hyposensitivity in the three major ABA responses (Supplementary Fig. 7a–d), demonstrating that the *cpn20* mutation is responsible for the observed ABA hypersensitive phenotypes.

We compared the *cpn20* mutants and *CPN20*-overexpressing lines with the widely known *abi5* mutant, *abi1*-*1* and *abi2*-*1* dominant mutants (Leung et al. 1997) and *abi1 abi2* double mutant in ABA responses of postgermination growth and stomatal movement. The *cpn20* mutants showed a similar intensity of ABA-hypersensitivity in ABA-induced postgermination growth arrest and promotion of stomatal closure compared to the *abi1 abi2* double mutant, though the *CPN20*-overexpressing lines were less resistant to ABA than the *abi5* mutant and *abi1*-*1* and *abi2*-*1* dominant mutants in ABA-induced postgermination growth arrest, and also less resistant to ABA than the *abi1*-*1* and *abi2*-*1* mutants in ABA-induced promotion of stomatal closure (Supplementary Fig. 8a–c).

We further tested whether two other chloroplast co-chaperonins CPN10(1) and CPN10(2), and the CPN60 chaperonin subunit CPN60α1, are involved in ABA signaling, and observed that none of the three proteins interacts with ABAR, and down-expression of CPN10(1), CPN10(2) or CPN60α1 does not alter ABA sensitivity (Supplementary Figs. 9–11), suggesting that CPN20 regulates ABA signaling independent of its cochaperonin nature.

### Down-expression of *CPN20* alters expression of a subset of ABA-responsive genes

**Fig. 3:**
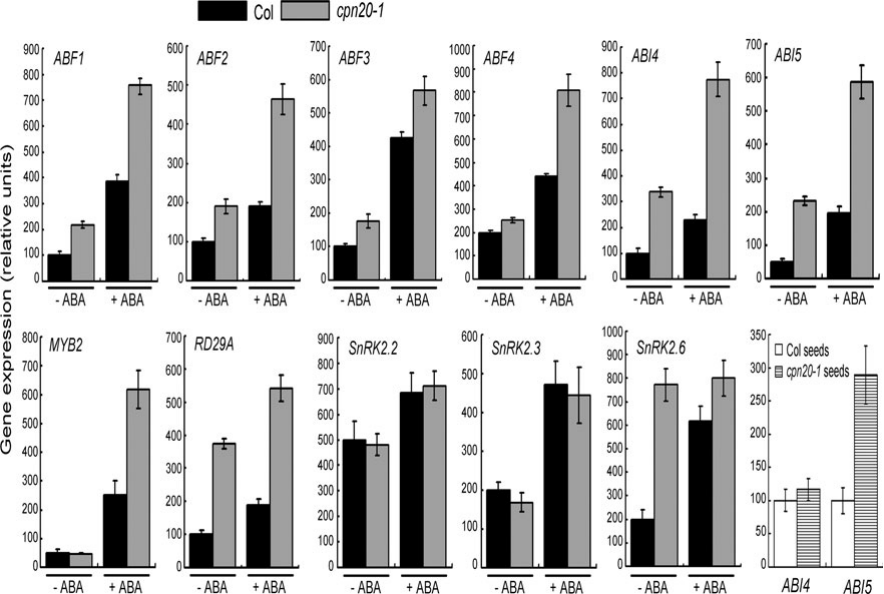
Analysis of gene expression. Expression of ABA-responsive genes in *cpn20*-*1* mutant. Gene expression was analyzed by real-time PCR. Seedlings of wild-type Col and *cpn20*-*1* mutant were transferred, 72 h after stratification, from ABA-free medium to medium supplemented with 0 μM or 1 μM of (±)ABA, and grown for 14 days before collected for analysis. *–ABA* 0 μM ABA treatment. *+ABA* 1 μM (±)ABA treatment. Expression of *ABI4* and *ABI5* was also assayed in the germinating seeds 24 h after stratification from wild-type Col and *cpn20*-*1* mutant. Each value is the mean ± SE of three biological determinations

We tested the expression of the following ABA-responsive genes in the *cpn20*-*1* mutant and the wild-type Col plants: *ABF1*, *ABF2*/*AREB1*, *ABF3*, *ABF4*/*AREB2* (Choi et al. 2000; Uno et al. 2000; Kang et al. 2002), *ABI4* (Finkelstein et al. 1998), *ABI5* (Finkelstein and Lynch 2000), *MYB2* (Abe et al. 2003), *RD29A* (Yamaguchi-Shinozaki and Shinozaki 1994), *SnRK2.2*, *SnRK2.3* and *SnRK2.6* (Fujii and Zhu 2009). Consistent with previous reports, expression of the most ABA-responsive genes was stimulated by the ABA treatment in wild-type plants (Fig. 3). Down-regulation of *CPN20* in *cpn20*-*1* mutant up-regulated expression of the most ABA-positive regulator-encoding genes: *ABF1*, *ABF2*, *ABF3*, *ABI4*, *ABI5*, *RD29A* and *SnRK2.6* regardless of absence or presence of ABA treatment (Fig. 3). Expression of *ABF4* and *MYB2* was strongly stimulated by ABA treatment in the *cpn20*-*1* mutant, though the expression levels of these two genes showed no significant difference between the *cpn20*-*1* mutant and wide-type plants in the absence of the ABA treatment (Fig. 3). However, down-expression of *CPN20* did not affect the expression of *SnRK2.2* and *SnRK2.3* genes (Fig. 3) involved in the PYR/PYL/RCAR ABA receptor-mediated signaling cascade (Cutler et al. 2010).

Given that *ABI4* and *ABI5* are expressed at a low level in seedlings after germination (Finkelstein et al. 1998, 2002; Finkelstein and lynch 2000; Lopez-Molina et al. 2001, 2002), we further assayed the expressions of these two genes in germinating seeds of the *cpn20*-*1* mutant and wild-type Col plants. We observed that the expression of *ABI5* was significantly up-regulated, but that of *ABI4* was not significantly changed in the *cpn20*-*1* mutant (Fig. 3), indicating that the effect of *CPN20* on *ABI4* expression depends on developmental stages. Taken together, these data support the negative role of CPN20 in ABA signal transduction, and suggest the complexity of the CPN20-mediated ABA signaling networks.

### Down-expression of *CPN20* suppresses ABA insensitivity of the *cch* mutant and the *ABAR*-RNAi lines

**Fig. 4:**
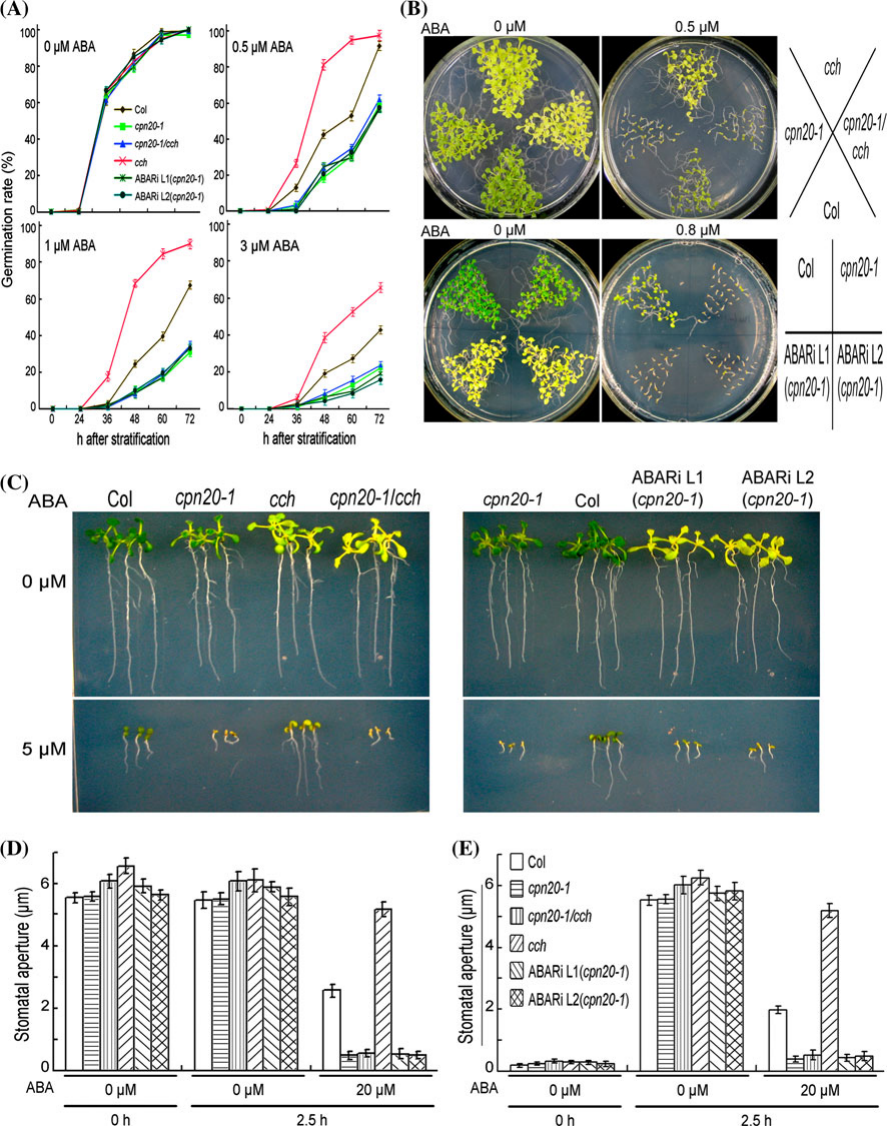
Genetic interaction between *CPN20* and *ABAR*: *cpn20* mutation suppresses *cch* and *ABAR*-RNAi mutant phenotypes. **a** Seed germination rates of *cpn20*-*1* and *cch* single mutant, *cpn20*-*1* *cch* double mutant and two *ABAR*-RNAi lines of *cpn20*-*1* mutant [indicated by ABARi L1 (*cpn20*-*1*) and ABARi L2 (*cpn20*-*1*)] in ABA-free (0 μM) and ABA-containing (0.5, 1 or 3 μM) medium at the indicated time points after stratification. Each value is the mean ± SE of three independent biological determinations. **b** Early seedling growth: seeds from the different genotypes (Col, mutants and transgenic lines) as mentioned in **a** were directly planted in the MS medium supplemented with different concentrations of (0.5 or 0.8 μM) (±)ABA and the observation was done 12 days after stratification. **c** Early seedling growth: germinating seeds of the different genotypes as mentioned in **a** were transferred, 48 h after stratification, from ABA-free medium to medium supplemented with 0 or 5 μM (±)ABA, and the growth was investigated 10 days after the transfer. **d** and **e** ABA-induced stomatal closure (**d**) and ABA-induced inhibition of stomatal opening (**e**) in the different genotypes as mentioned in **a**. Values are the mean ± SE of five independent experiments; n = 60 apertures per experiment

Previous study showed that the *cch* mutant, an allele of *gun5* with a single amino acid mutation Pro642 → Leu, has the ABA-insensitive phenotypes in seed germination, seedling growth and ABA-induced stomatal movement, like the transgenic *ABAR*-RNAi (RNA interference) lines (Shen et al. 2006; Wu et al. 2009; Shang et al. 2010; Du et al. 2012). We generated *cch* *cpn20*-*1* double mutant, and observed that the double mutant showed ABA hypersensitivity in all the major ABA responses including ABA-induced seed germination inhibition, postgermination growth arrest, and ABA-induced stomatal closure and inhibition of stomatal opening (Fig. 4a–e; Supplementary Fig. 5b). We further introduced the *ABAR*-RNAi into the *cpn20*-*1* mutant and similar ABA-hypersensitive phenotypes were observed for all these transgenic *ABAR*-RNAi-*cpn20*-*1* lines (Fig. 4a–e; Supplementary Figs. 5d, 12a, 13), while the transgenic *ABAR*-RNAi lines of wild-type Col background (a control) showed ABA-insensitive phenotypes (Supplementary Fig. 13). These data showed that the knockdown mutation of *CPN20* gene is epistatic to *cch* point mutation or RNAi-down-expression mutation of *ABAR* gene, suggesting that CPN20 functions downstream of ABAR in ABA signaling pathway.

### *ABAR*-overexpression does not change *cpn20* mutant phenotypes and *CPN20*-overexpression does not modify *cch* mutant phenotypes either

**Fig. 5:**
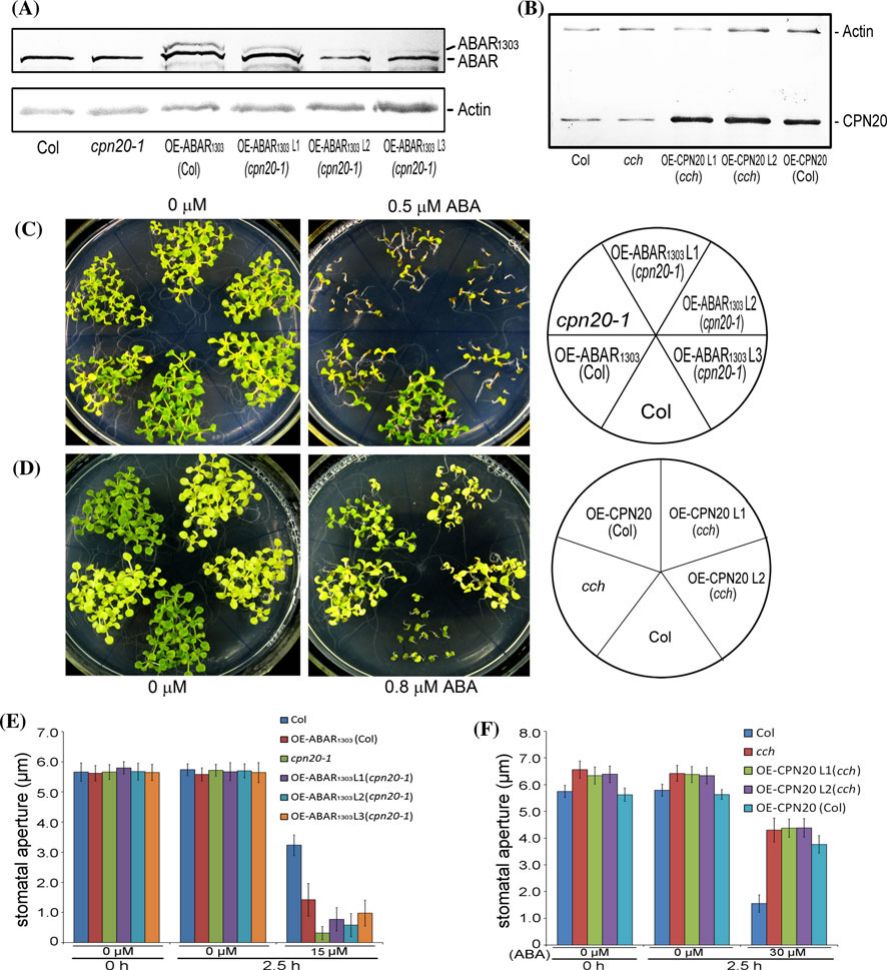
Genetic interaction between *CPN20* and *ABAR*: *ABAR*-overexpression does not affect *cpn20* mutant phenotypes and *CPN20*-overexpression does not modify *cch* mutant phenotypes either. **a** and **b** Immunoblotting analysis of the *ABAR*-overexpression lines under wild-type Col [OE-ABAR_1303_(Col)] or *cpn20*-*1* mutant background [OE-ABAR_1303_ L1/L2/L3(*cpn20*-*1*)] (**a**) and the *CPN20*-overexpression lines under wild-type Col [OE-CPN20(Col)] or *cch* mutant background [OE-CPN20 L1/L2(*cch*)] (**b**). ABAR_1303_ is a truncated form of ABAR (amino acid residues 1–1303), whose expression results in ABA-hypersensitive phenotypes (Wu et al. 2009). Two ABAR forms, one ABAR_1303_ linked to GFP (the top bands, a) and another full-length natural ABAR (the bottom bands, a), were immuno-detected in the ABAR_1303_-overexpression lines (**a**). The Over-expressed amounts of CPN20 were detected in the *CPN20*-overexpression lines under the Col/cch background compared to those in the Col/*cch* (**b**). Actin is used as a loading control. The experiments were repeated three times with similar results. **c** Early seedling growth of the different genotypes described in **a**. Seeds were directly planted in the ABA-free medium or the medium supplemented with 0.5 μM (±)ABA and the observation was done 12 days after stratification. **d** Early seedling growth of the different genotypes described in **b**. Seeds were directly planted in the ABA-free medium or the medium supplemented with 0.8 μM (±)ABA and the observation was done 12 days after stratification. **e** ABA-induced stomatal closure in the different genotypes as mentioned in **a**. **f** ABA-induced stomatal closure in the different genotypes as mentioned in **b**. Values in **e** and **f** are the mean ± SE of five independent experiments; n = 60 apertures per experiment

We further created *ABAR*-overexpression lines under *cpn20*-*1* mutant background (OE-ABAR-*cpn20*) by crossing *cpn20*-*1* mutant with an *ABAR*-overexpression line under wild-type Col background (OE-ABAR), in which an ABAR truncated form, ABAR_1303_ (amino acid residues 1–1303) linked to GFP, was expressed (Fig. 5a). The expression of this truncated form in wild-type plants has been shown to result in ABA-hypersensitive phenotypes (Wu et al. 2009). We also created *CPN20*-overexpression lines under *cch* mutant background (OE-CPN20-*cch*) by crossing *cch* mutant with a *CPN20*-overexpression line under wild-type Col background (OE2 as described earlier, here named OE-CPN20) (Fig. 5b, d, f). We observed that the OE-ABAR-*cpn20* lines displayed ABA-hypersensitive phenotypes more like *cpn20*-1 mutant than the OE-ABAR line (Fig. 5c, e), consistent with the above-described genetic epistasis of CPN20 to ABAR. Interestingly, however, we further observed that the OE-CPN20-*cch* lines showed ABA-insensitive phenotypes similar to their parental *cch* mutant than the OE-CPN20 line especially in ABA-induced stomatal closure (Fig. 5d, f). These data suggest that the function of CPN20 also requires functional ABAR, and indicate a possibility that CPN20 functions at the same node as ABAR in the ABA signaling pathway.

### The *wrky40* mutation suppresses ABA-insensitive phenotypes of *CPN20*-overexpression lines

**Fig. 6:**
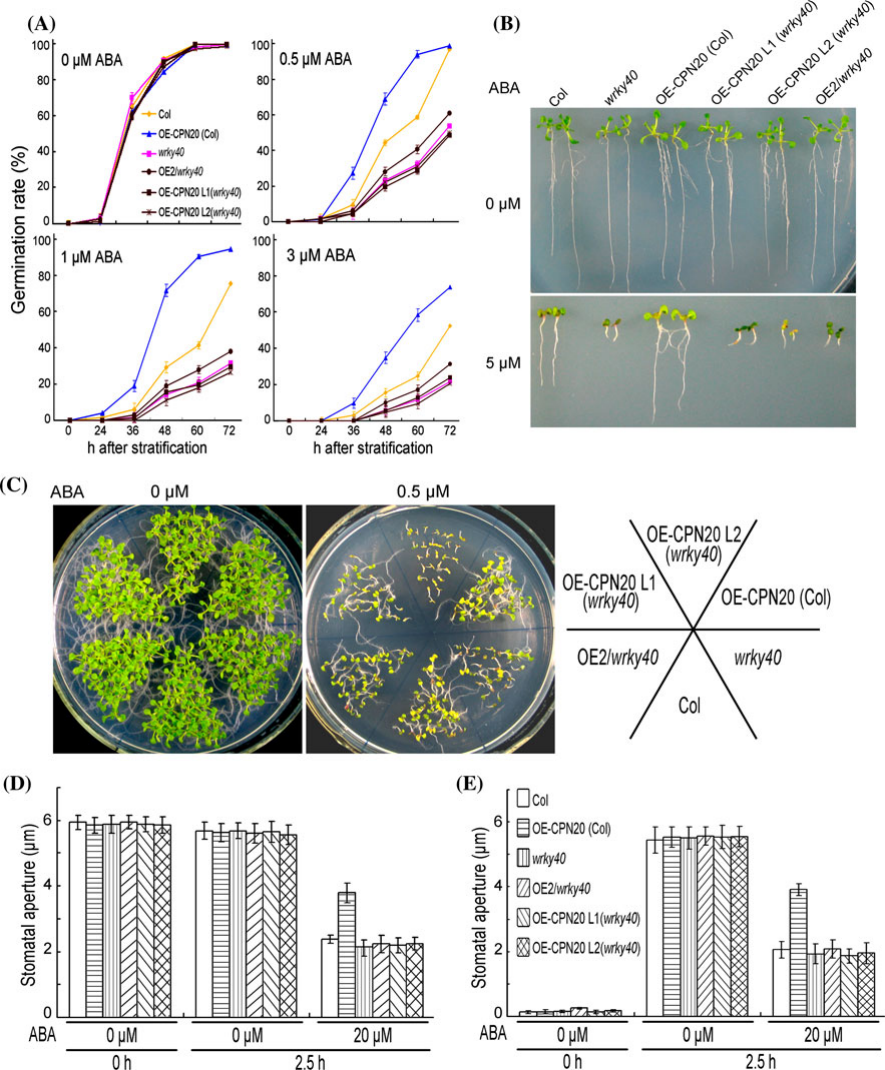
Genetic interaction between *CPN20* and *WRKY40*: *wrky40* mutation suppresses ABA-insensitive phenotypes of *CPN20*-overexpression lines. **a** Seed germination rate of the *wrky40* mutant, a *CPN20*-overexpressing line of the Col background [OE-CPN20 (Col)], two *CPN20*-overexpressing lines of the *wrky40* mutant background [OE-CPN20 L1 (*wrky40*), OE-CPN20 L2 (*wrky40*)], and OE-CPN20 *wrky40* double mutant in ABA-free (0 μM) and ABA-containing (0.5, 1 or 3 μM) medium at indicated time points after stratification. Each value is the mean ± SE of three biological determinations. **b** and **c** Early seedling growth of the different genotypes as mentioned in **a**. **b** Seedlings were transferred, 48 h after stratification, from ABA-free medium to the medium supplemented with 0 or 5 μM (±)ABA, and image was taken 10 days after the transfer. **c** Seeds were directly planted in the MS medium supplemented with 0 or 0.5 μM (±)ABA, and the growth was investigated 12 days after stratification. **d** and **e** ABA-induced stomatal closure (**d**) and ABA-inhibited stomatal opening (**e**) in the different genotypes as described in **a**. Values are the mean ± SE of five independent experiments; n = 60 apertures per experiment

We previously showed that disruption of WRKY40 in the *wrky40* mutant causes ABA hypersensitive phenotypes in ABA-induced inhibition of seed germination and post-germination growth arrest (Shang et al. 2010; Liu et al. 2012; Yan et al. 2012). Since CPN20 is another interaction partner of ABAR in addition to WRKY40, we tested whether and how CPN20 interacts with WRKY40 genetically. The *wrky40*, a knockout mutant allele of the *WRKY40* gene was used in this experiment. Given that both *cpn20* and *wrky40* mutants are ABA hypersensitive, which causes difficulties to compare the single *cpn20* or *wrky40* mutant with *cpn20* *wrky40* double mutant in ABA hypersensitivity, we created *CPN20*-overexpression lines in the *wrky40* mutant background, and crossed the OE2 (the *CPN20*-overexpression line 2 in Col background) with *wrky40* mutant to obtain the *wrky40* mutant lines where *CPN20* is over-expressed (Supplementary Fig. 12b). Phenotypic analysis showed that all the transgenic-mutant lines harboring overexpressed-*CPN20* coupled with disruption of *WRKY40* displayed the ABA hypersensitive phenotypes in seed germination and early seedling growth, which were similar to the *wrky40* loss-of-function mutant (Fig. 6a–c; Supplementary Fig. 5c). Further, we observed that the introduction of the *wrky40* mutation into the *CPN20*-overexpression lines suppressed ABA-insensitivity in stomatal movement of the *CPN20*-overexpression lines (Fig. 6d, e). These data support the idea that CPN20 functions upstream of WRKY40 in ABA signaling pathway.

## Discussion

### CPN20 negatively regulates ABA signaling independently of its co-chaperonin function

The chloroplast CPN20 plays an essential function in the assistance of protein folding mediated by CPN60 in plant chloroplasts (Bertsch et al. 1992). Previous reports suggested that CPN20 may also function to regulate cellular processes independent of its co-chaperonin role (Weiss et al. 2009) essentially according to the high expression level of *CPN20* gene (Peltier et al. 2006; Weiss et al. 2009), and a most recent study supported an independent role of CPN20 in the regulation of the iron superoxide dismutase activity in the *Arabidopsis* chloroplasts (Kuo et al. 2013). In the present study in *Arabidopsis*, we observed that down-regulation of the *CPN20* gene enhances, but up-regulation of the *CPN20* gene reduces, ABA sensitivity in all the major ABA responses including ABA-inhibited seed germination, ABA-induced post-germination growth arrest, ABA-induced stomatal closure and ABA-inhibited stomatal opening, and plant tolerance to water stress, and also alters a set of ABA-responsive genes (Figs. 2, 3; Supplementary Figs. 4–8). These data reveal that the *Arabidopsis* CPN20 is negatively involved in ABA signaling. In contrast, however, two other chloroplasts co-chaperonins CPN10(1) and CPN10(2), both of which function, like CPN20, in the process of protein folding together with the CPN60 (Hill and Hemmingsen 2001; Koumoto et al. 2001), are not involved in ABA signaling (Supplementary Fig. 11). CPN60 does not regulate ABA signaling either (Supplementary Fig. 12). Additionally, neither CPN10(1)/CPN10(2) nor CPN60α1 interacts with ABAR (Supplementary Figs. 9, 11), excluding the possibility that these chaperonin and co-chaperonins act on ABAR to modulate ABA signaling. These data demonstrate that CPN20 functions as a negative regulator of ABA signal transduction, and provides new evidence that CPN20 mediates cellular processes independently of its co-chaperonin function.

### How does CPN20 work in ABA signaling?

Previous studies showed that ABAR antagonizes the WRKY40 transcription repressor, a negative ABA signaling regulator, which inhibits a subset of ABA-responsive genes involved in ABA-induced physiological responses (Shang et al. 2010; Liu et al. 2012; Yan et al. 2012). The present study identified CPN20 as another interaction partner of ABAR, which is, like WRKY40, negatively involved in ABA signaling. We provided genetic evidence demonstrating that CPN20 functions downstream or at the same node of ABAR (Figs. 4, 5) but upstream of WRKY40 transcription repressor in ABA signaling pathway (Fig. 6). These findings link CPN20 to the ABAR-WRKY40 coupled signaling cascades. However, how CPN20 functions to link ABAR and WRKY40 in ABA signaling is an open question. We hypothetically suggest that CPN20 may positively regulate WRKY40 expression by interacting with and antagonizing ABAR. CPN20 and ABAR may cooperate to function in ABA signaling, consequently leading to repression of the WRKY40 transcription repressor in response to ABA, which finally induces ABA-related physiological responses. Further studies will be needed to explore the unknown signaling mechanisms in these signaling cascades.

## Electronic supplementary material

Below is the link to the electronic supplementary material.

Supplementary material 1 (PDF 1896 kb)
