# Supplementary material for: Cochaperonin CPN20 negatively regulates abscisic acid signaling in Arabidopsis
Source: Plant Mol Biol. 2013 Jun 20;83(3):205–18. doi: 10.1007/s11103-013-0082-8 (PMC3777161; doi:10.1007/s11103-013-0082-8)
Supplement: Supplementary file 1 — Supplementary material 1 (PDF 1896 kb) [file 11103_2013_82_MOESM1_ESM.pdf]

Supplemantary Materials

Zhang XF et al.: Cochaperonin CPN20 negatively regulates abscisic acid signaling in *Arabidopsis*

Supplementary Materials and Methods

CPN20-Promoter-GUS Transgenic Assay

The promoter fragment of the *CPN20* gene was cloned into the pCAMBIA1391 vector using the primers listed in Supplemental Table 1. The construct was introduced into *A. tumefaciens* strain GV1301 for transformation of *Arabidopsis* (Col-0) plants by floral infiltration. Hygromycin-resistant transgenic seedlings of T3 generation were used for the analysis of GUS activity. For GUS staining, whole plants or tissues were immersed for 6 hours at 37℃ in 1 mM 5-bromo-4-chloro-3-indolyl-β-GlcUA (X-gluc) solution in 100 mM sodium phosphate (pH 7.0), 2 mM EDTA, 0.05 mM ferricyanide, 0.05 mM ferrocyanide and 0.1% (V/V) Trion X-100. Chlorophyll was cleared from the tissues with a mixture of 30% acetic acid and 70% ethanol.

Transient Expression in Arabidopsis Protoplasts

CPN20 was tagged by GFP or mCherry, and ABAR was tagged by GFP for transient expression in the *Arabidopsis* protoplasts. The chloroplast stroma (RbcS; Lee et al., 2002) marker was tagged by mCherry for observation of precise localization of CPN20. Primers used in this experiment are listed in Supplemental Table 1. Protoplasts were isolated from the rosette leaves of 4-week old plants (ecotype Col-0) and transiently transformed using polyethylene glycol (PEG) essentially according to Sheen's protocol (http://genetics.mgh.harvard.edu/sheenweb/). The protoplasts co-transformed by *CPN20-GFP* and marker gene-mCherry, or co-transformed by *CPN20-mCherry* and *ABAR-GFP* were incubated at 23℃ for 16 hours. Fluorescence of GFP or mCherry was detected with a confocal laser scanning microscope (Zeiss LSM 510 META). The GFP fluorescence and mCherry fluorescence were excited with a 488 nm and 543 nm argon-ion laser, filtered with 545 nm spectroscopes. The GFP fluorescence was detected with a 505-530 nm filter set and the mCherry fluorescence was detected with a 585-615 nm filter set.

Production of anti-CPN20 serum

The full-length cDNA of *CPN20* was isolated using the primers listed in Table S1. To produce glutathione S-transferase (GST) tag fusion protein, *Escherichia coli* DH5α cells transformed with the pGEX-4T-1-CPN20 construct were induced with 0.2 mM IPTG for 16 hours at 16℃. The fusion protein was purified from IPTG-induced cell pellets by a glutathione-Sepharose 4B column (Amersham Pharmacia Biotech) and analyzed by SDS-PAGE. The purified fusion protein was used for standard immunization protocols in rabbits. Polyclonal anti-serum obtained was affinity-purified by HiTrap Protein-A HP (Amersham Pharmacia Biotech). The affinity-purified anti-serum was evaluated by immunoblotting and shown to be highly specific to CPN20.

CoIP assays in yeast and in planta

CoIP assays were performed using extracts of both yeast cells and *Arabidopsis* plants. For immunoprecipitation in yeast, yeast strains were grown on SD4- medium to OD_{600} 1.0 at 30℃. Total protein was prepared from yeast cells with an extraction buffer (2 mL/g cells) containing 50 mM HEPES, pH 7.4, 10 mM EDTA, 0.1% (V/V) Triton X-100, 1 mM PMSF, and 1 μg/mL each of aprotinin, leupeptin and pepstatin A (Sigma-Aldrich).

Immunoprecipitation experiments were performed following the manufacturer's protocol. Cell lysates were pre-cleared with protein A/G Plus-agarose beads (Santa Cruz Biotechnology) and incubated with the anti-HA serum and the protein A/G Plus-agarose beads at 4℃ overnight in the extraction buffer. The beads were washed twice extensively with buffer A containing 50 mM Tris (pH 8.0), 150 mM NaCl, and 0.1% (V/V) Triton X-100 and buffer B containing 50 mM Tris (pH 8.0), and 0.1 % (V/V) Triton X-100 and then re-suspended in SDS-PAGE sample buffer. The immuno-precipitates were separated on a 12% SDS-PAGE and analyzed by immunoblotting with anti-MYC serum. For immunoprecipitation in *Arabidopsis* extracts, the total protein was re-suspended in the extraction buffer (3 mg/mL) containing 50 mM Tris-HCl, pH 7.4, 150 mM NaCl, 1 mM EDTA, 0.1 % (V/V) Triton X-100, 10% (V/V) glycerol, 1 mM PMSF, and 1 µg/mL cocktail (Merck). The immunoprecipitation was done with the same procedures as described above except that the anti-ABAR or anti-CPN20 serum was used instead of the anti-Myc or anti-HA serum, and the beads were washed with the extraction buffer instead of the buffer A and buffer B.

Luciferase complementation imaging (LCI)

Luciferase complementation imaging (LCI) assay was used to detect protein-protein interaction in *N. benthamiana* leaves according to previously described procedures (Chen et al., 2008; Shang et al., 2010). The firefly Luc enzyme is divided into the N-terminal part (NLuc) and C-terminal part (CLuc). ABAR was fused with NLuc in pCAMBIA-NLuc vector, and CPN20, CPN20ΔNS or WRKY40 was fused with CLuc in pCAMBIA-CLuc vector respectively. Primers used for the vector construction were shown in Table S1. The constructs were mobilized into *A. tumefaciens* GV3101. Bacteria were suspended in infiltration buffer (0.2 mM acetosyringone, 10 mM $MgCl_2$, and 10 mM MES) to identical concentrations (OD_{600} = 0.6). Equal concentrations and volumes of bacteria were mixed and co-infiltrated into the 7-week-old *N. benthamiana* leaves using needleless syringes. After infiltration, plants were placed with 16 h-light/8 h-dark for 48 h at 24℃. The Luc activity was observed with a low-light cooled CCD imaging apparatus (Andor iXon). The mouse anti-full-length firefly Luc antibody (Santa Cruz Biotechnology) was used to immunodetect Luc fusion protein in transgenic tissues. All experiments were repeated at least five independent biological replicates.

Real-time PCR analysis

Total RNA was isolated from 10 day-old seedlings using a Total RNA Rapid Extraction Kit (BioTeke), treated with RNase-free DNase I (TAKARA) at 37℃ for 30 min to degrade genomic DNA and purified by using an RNA Purification Kit (BioTeke). A 2-µg aliquot of RNA was subjected to first-strand cDNA synthesis using M-MLV reverse transcriptase (Promega), and an oligo (dT21) primer. The primers of various ABA-responsive genes used for real-time PCR are listed in Table S1. Analysis was performed using the BioRad Real-Time System CFX96TM C1000 Thermal Cycler (Singapore). All experiments were repeated at least three times along with three independent repetitions of the biological experiments.

Phenotypic analysis

For the germination assay, about 100 seeds each from wild-type plants and mutants or transgenic lines were sterilized and planted in triplicate on MS medium. The medium contained 3% sucrose and 0.8% agar (pH5.9) and was supplemented with or without different concentrations of (±)ABA. The seeds were incubated at 4℃ for 3 days and placed at 21℃ under light conditions. Germination (emergence of radicals) was scored at the indicated times. For the seedling growth assay, the seeds were planted directly in ABA-containing MS medium or transferred 48 hours after stratification from the common MS medium to medium supplemented with different concentrations of ABA. Images were taken after 12 to14 days.

For stomatal aperture assay, rosette leaves were used. To observed ABA-induced stomatal closure, leaves were immersed in solution containing 50 mM KCl and 10 mM MES-KOH (pH6.15) and exposed to a halogen cold light

source for 3 hours. Subsequently, different concentrations of (±)ABA were added to the solution. Stomatal apertures were measured after 2.5 hours' ABA treatment. To study ABA-inhibited stomatal opening, leaves were immersed in the same solution in the dark for 3 hours before they were transferred to the cold light for 2.5 hours in the presence of ABA, and then apertures were recorded. For water loss assay, rosette leaves were detached and placed on filter paper. Water loss was evaluated by weighing excised leaves at the indicated times. For drought treatment, plants were grown on soil for about 2 weeks, and then drought was imposed by withdrawing irrigation until the lethal effect of dehydration was observed on the majority of the plants. Plants grown under a standard irrigation regime were used as a control.

Accession Numbers

Sequence data from this article can be found in the Arabidopsis Genome Initiative database under the following accession numbers: *CPN20* (AT5G20720), *CPN10(1)* (AT3G60210), *CPN10(2)* (AT2G44650), *CPN60α1*(AT2G28000), *ABAR*/*CHLH* (At5g13630), *WRKY40* (At1g80840) and *WRKY18* (At4g31800). Germplasm identification numbers for mutant lines are as follows: *cpn20*-1 (SAIL_888_A09), *cpn20*-2 (SALK_083054C), *cpn20*-3 (CR360869), *wrky40*-1 (stock number: ET5883, Cold Spring Harbor Laboratory gene and enhancer trap lines), *cpn10(1)*-1 (SALK_093761), *cpn10(1)*-2 (SALK_027792C), *cpn10(2)*-1 (SALK_023440), *cpn10(2)*-2 (SALK_051544), and *cpn60α1*(SALK_082308).

Supplementary Results

Downregulation of CPN10(1), CPN10(2) or CPN60α1 does not alter ABA sensitivity

We tested whether two other chloroplast co-chaperonins, CPN10(1) and CPN10(2), are involved in ABA signaling. A yeast two-hybrid assay showed that neither of the two CPN10s interacts with ABAR (Fig. S9). We further showed that disruption or downregulation of the *CPN10(1)* and *CPN10(2)* genes did not affect the three major ABA responses (Fig. S10), which demonstrates that CPN10(1)/(2) are not involved in ABA signaling.

We further tested whether the interaction partner of CPN20, CPN60 chaperonin, is involved in ABA signaling. Plant CPN60 includes two classes of subunits, CPN60α and CPN60β (Hill and Hemmingsen, 2001), in which *Arabidopsis* CPN60α is composed of two homologues, CPN60α1 and CPN60α2, and CPN60β of four homologues, CPN60β1, CPN60β2, CPN60β3 and CPN60β4 (Hill and Hemmingsen, 2001). These CPN60 subunits form different complex to function as molecular chaperons (Bonshtien et al., 2009). Previous reports showed that *CPN60α1* is expressed at higher level than *CPN60α2* (Weiss et al., 2009), and disruption mutation of *CPN60α1* is lethal (Suzuki et al., 2009). We screened one T-DNA insertional knock-down mutant of *CPN60α1* gene (SALK_082308), named *cpn60α1*, which shows developmental defects such as small size of seedlings and advanced flowering time (Fig. S11, A-C), but wild-type ABA responses (Fig. S11, D and E). We could not measure stomatal response to ABA in this mutant because of its too small size of mature leaves (Fig. S11C). In addition, we observed that CPN60α1 does not interact with ABAR (Fig. S11F). Given that the different α and β subunits of CPN60 form complexes to execute their functions in cells (Bonshtien et al., 2009), these data support the idea that CPN60 is not involved in ABA signaling.

Supplementary References

Bonshtien AL, Parnas A, Sharkia R, et al. Differential effects of co-chaperon in homologs on cpn60 oligomers. *Cell Stress Chaperones* 2009; **14:** 509-519.

Chen H, Zou Y, Shang Y, et al. Firefly luciferase complementation imaging assay for protein-protein interactions in plants. *Plant Physiol* 2008; **146**: 368-376.

Hill JE, Hemmingsen SM. *Arabidopsis thali*ana type I and II chaperonins. *Cell Stress Chaperones* 2001; **6**: 190-200.

Lee KH, Kim DH, Lee SW, Kim ZH, Hwang I. *In vivo* import experiments in protoplasts reveal the importance of the overall context but not specific amino acid residues of the transit peptide during import into chloroplasts. *Mol Cell* 2002; **14**: 388-397.

Shang Y, Yan L, Liu ZQ, et al. The Mg-chelatase H subunit antagonizes a group of WRKY transcription repressors to relieve ABA-responsive genes of inhibition. *Plant Cell* 2010; **22**: 1909-1935.

Suzuki K, Nakanishi H, Bower J, Yoder DW, Osteryoung KW, Miyagishima S**.** Plastid chaperonin proteins Cpn60α and Cpn60β are required for plastid division in *Arabidopsis thaliana*. *BMC Plant Biol* 2009; **9**: 38.

Weiss C, Bonshtien A, Farchi-Pisanty O, Vitlin A**.** Cpn20: Siamese twins of the chaperonin world. *Plant Mol Biol* 2009; **69**: 227-238.

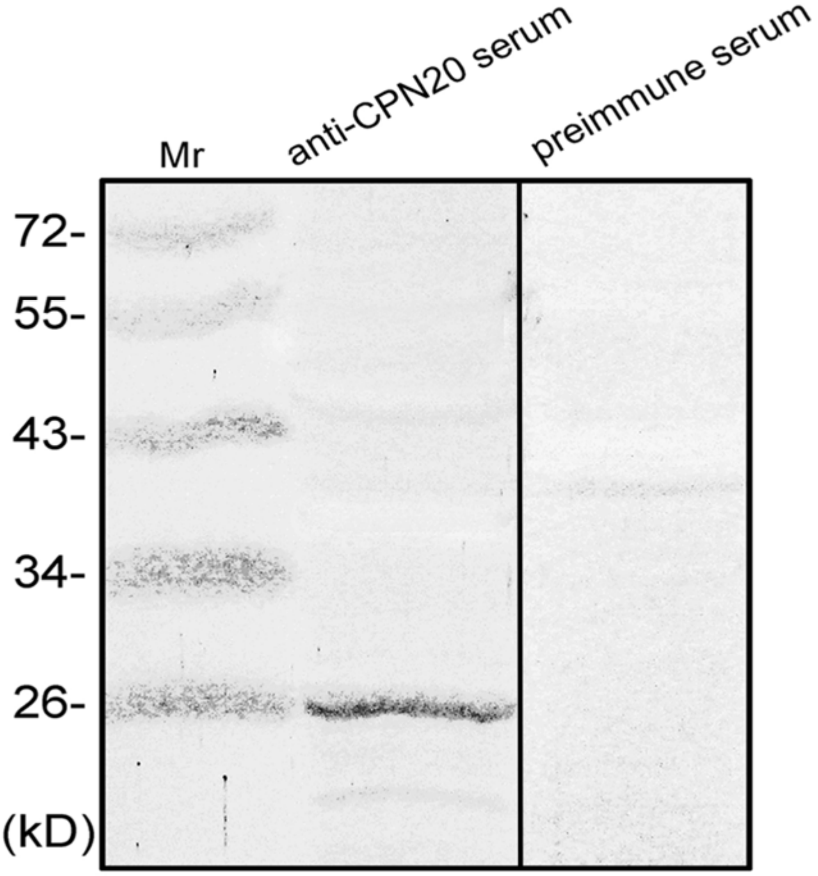

Supplementary Fig. 1. Test of the Specificity of the Anti-CPN20 Serum.
Arabidopsis total protein was immuno-blotted by anti-CPN20 serum and preimmune serum, showing that the anti-CPN20 serum recognizes specifically a 26-kD protein (CPN20). Mr, molecular mass markers; kD, kilodalton.

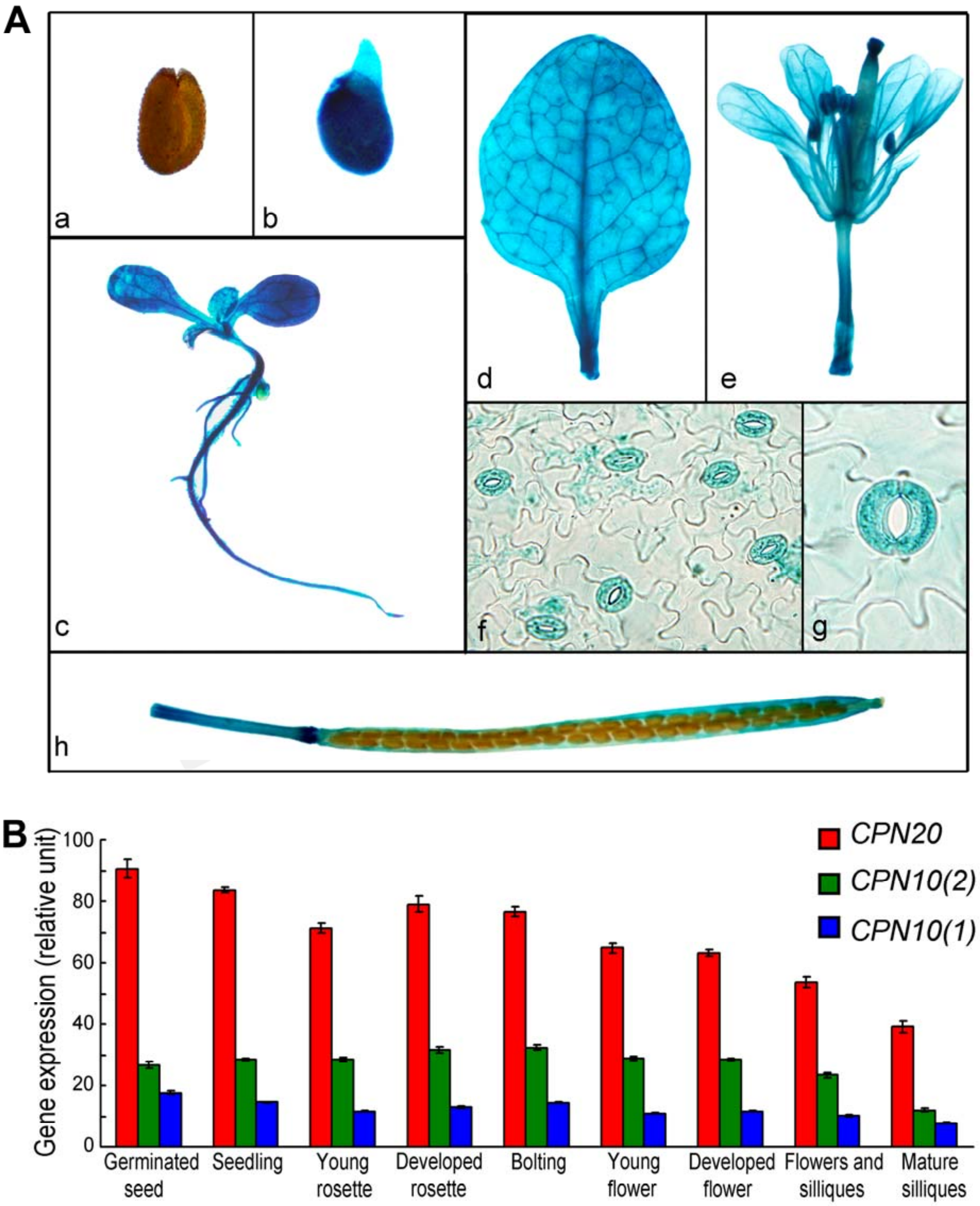

Supplementary Fig. 2. Expression Profile of *CPN20* Gene.
(A) Expression of *CPN20* in different organs/tissues. *CPN20* is expressed ubiquitously in different tissues/organs, except for dry seeds. The expression was analyzed by the *CPN20*-promoter-linked glucuronidase (GUS)-transgenic plants. **(a)** Dry seed. **(b)** Germinated seed 24 h after stratification. **(c)** Young seedling 10 days after stratification. **(d)** Rosette leaf. **(e)** Flower. **(f)** and **(g)** Stoma. **(h)** Silique. All the transgenic lines showed the same expression pattern.
(B) Expression of *CPN20* (red columns) and its two homologous genes, *CPN10(1)* (blue columns) and *CPN10(2)* (green columns), in different tissues/organs according to the data published online at the website Genevestigator (http://www.genevestigator.com).

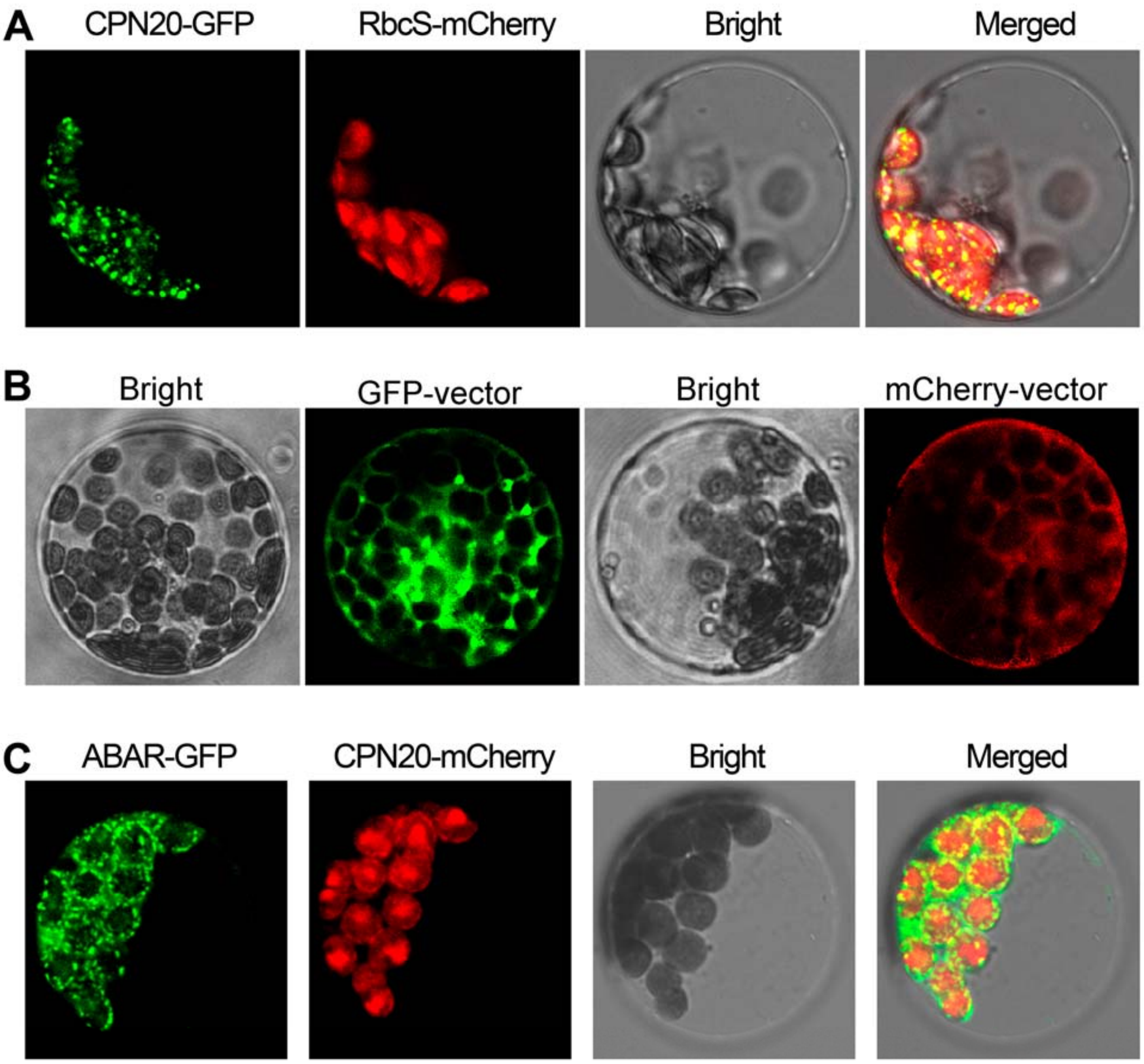

Supplementary Fig. 3. Subcellular Localization of CPN20.
(A) Transient co-expression of the CPN20-GFP fusion protein with a chloroplast stroma marker RbcS-mCherry (Lee et al., 2002) in *Arabidopsis* protoplasts: CPN20 is expressed in the chloroplast stroma together with RbcS-mCherry. Bright, bright field; Merged, merged image of CPN20-GFP and RbcS stroma marker in bright field. The experiments were repeated three times with the same results. **(B)** Control images for GFP vector and mCherry vector: the green or red fluorescence are mainly distributed in cytosolic space, which is different from that of GFP-tagged CPN20 or mCherry-tagged chloroplast markers. The experiments were repeated three times with the same results. **(C)** Transient expression in *Arabidopsis* protoplasts shows that CPN20-mCherry and ABAR-GFP partly co-localize into the stroma and especially peripheral areas of the chloroplast (yellow areas). Bright, bright field; Merged, merged image of CPN20-mCherry and ABAR-GFP in bright field. The experiments were repeated three times with the same results.

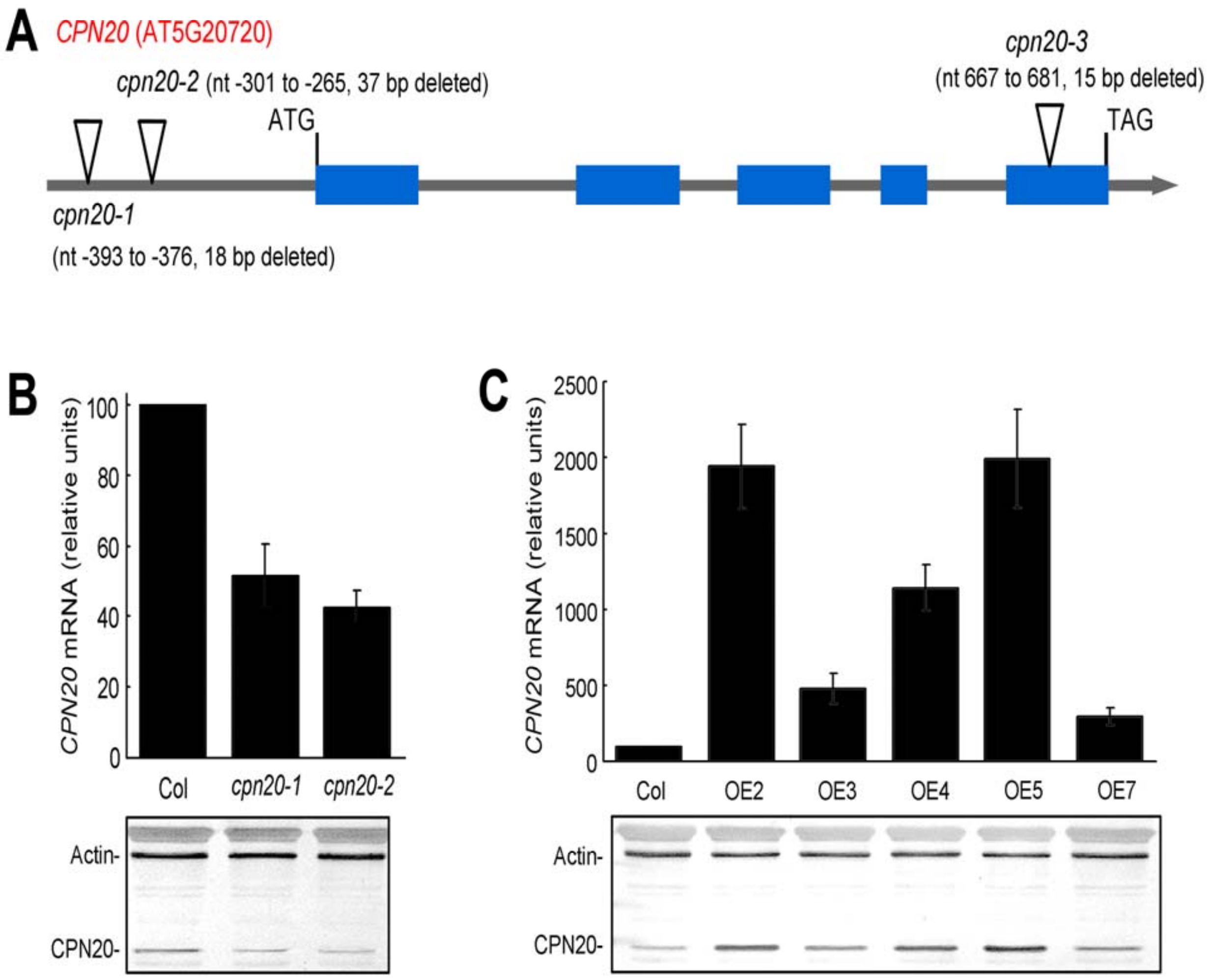

Supplementary Fig. 4. Identification of the *cpn20* Mutants and *CPN20*-Overexpression Lines.
(A) T-DNA insertion sites in the three mutant alleles *cpn20-1*, *cpn20-2* and *cpn20-3* (with Col ecotype as background). Blue boxes and grey lines represent exons and introns, respectively. The T-DNA insertion sites are indicated by open arrowheads, and nt represents nucleotides. In the *cpn20-1* mutant allele, the T-DNA was inserted into the promoter region at nt -393 to -376 upstream of the start codon ATG of *CPN20* with an 18-bp fragment deleted. In the c*pn20-2* mutant allele, the T-DNA was inserted into the promoter region at nt -301 to -265 upstream of the start codon ATG of *CPN20* with a 37-bp fragment deleted. In the *cpn20-3* mutant, the T-DNA was inserted into the fifth exon at nt 667 to 681 downstream of the start codon ATG of *CPN20* with a 15-bp fragment deleted. **(B)** and **(C)** Real-time PCR and immunoblotting analysis of *CPN20* expression in wild-type Col, homozygous mutants *cpn20-1* and *cpn20-2*, and five CPN20-overexpression lines (OE2, OE3, OE4, OE5, and OE7). For the real-time PCR analysis, the value obtained from the 4-week old Col seedling after stratification was taken as 100%, and all the other values were normalized relative to this value. Each value for real-time PCR is the mean ± SE of three independent biological determinations. Immunoblotting was performed with anti-CPN20 serum in the total protein extracted from the leaves of the seedlings grown for 4 weeks after stratification. Actin was used as a loading control. The immunoblotting assays were repeated three times with the independent biological experiments, which gave the similar results.

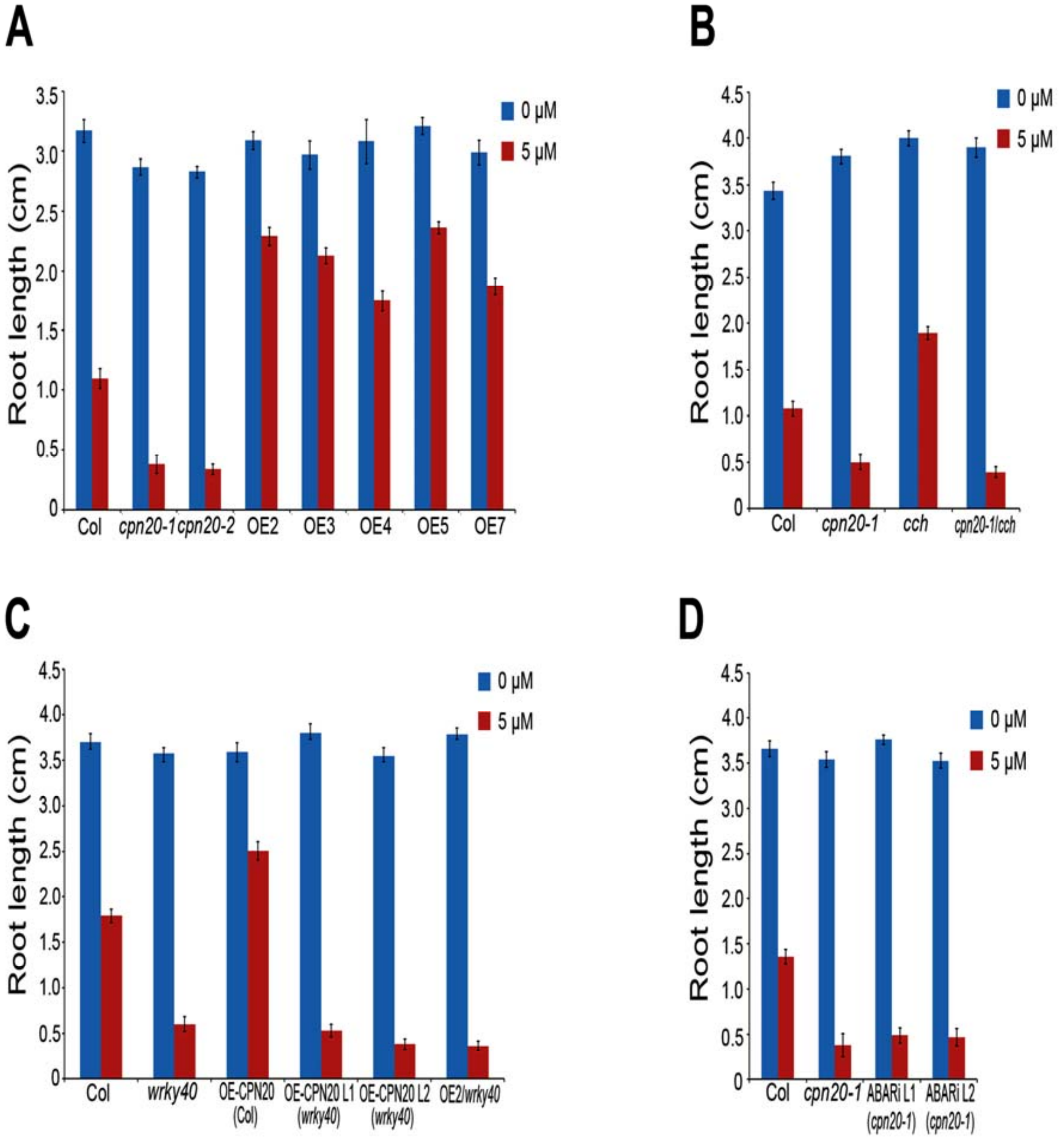

Supplementary Fig. 5. Quantification of Root Growth in Different Mutants and Transgenic Lines. Seedlings were transferred from the ABA-free medium to the medium supplemented with 0 or 5 µM (±)ABA, and the primary root length was measured 10 days after the transfer. **(A)** The statistics for the *cpn20* mutants and *CPN20*-overexpression transgenic lines as described in **Fig. 2C**. **(B)** The statistics for the *cpn20-1*, *cch* single mutants and *cpn20-1 cch* (*cpn20*/*cch*) double mutant as described in **Fig. 4C**. **(C)** The statistics for the *wrky40* mutant, the *CPN20*-overexpression (OE-CPN20) line in the Col background [OE-CPN20 (Col)], the OE-CPN20 lines in the *wrky40* mutant background (OE-CPN20 (*wrky40*)) and OE-CPN20 *wrky40* double mutant (OE-CPN20/*wrky40*) as described in **Fig. 6B**. **(D)** The statistics for the *cpn20-1* mutant and *ABA*R-RNAi lines in the *cpn20-1* mutant background [ABARi L1/L2 (*cpn20-1*)] as described in **Fig. 4C**. Each value in **(A)** to **(D)** is the mean ±SE of at least 30 seedlings.

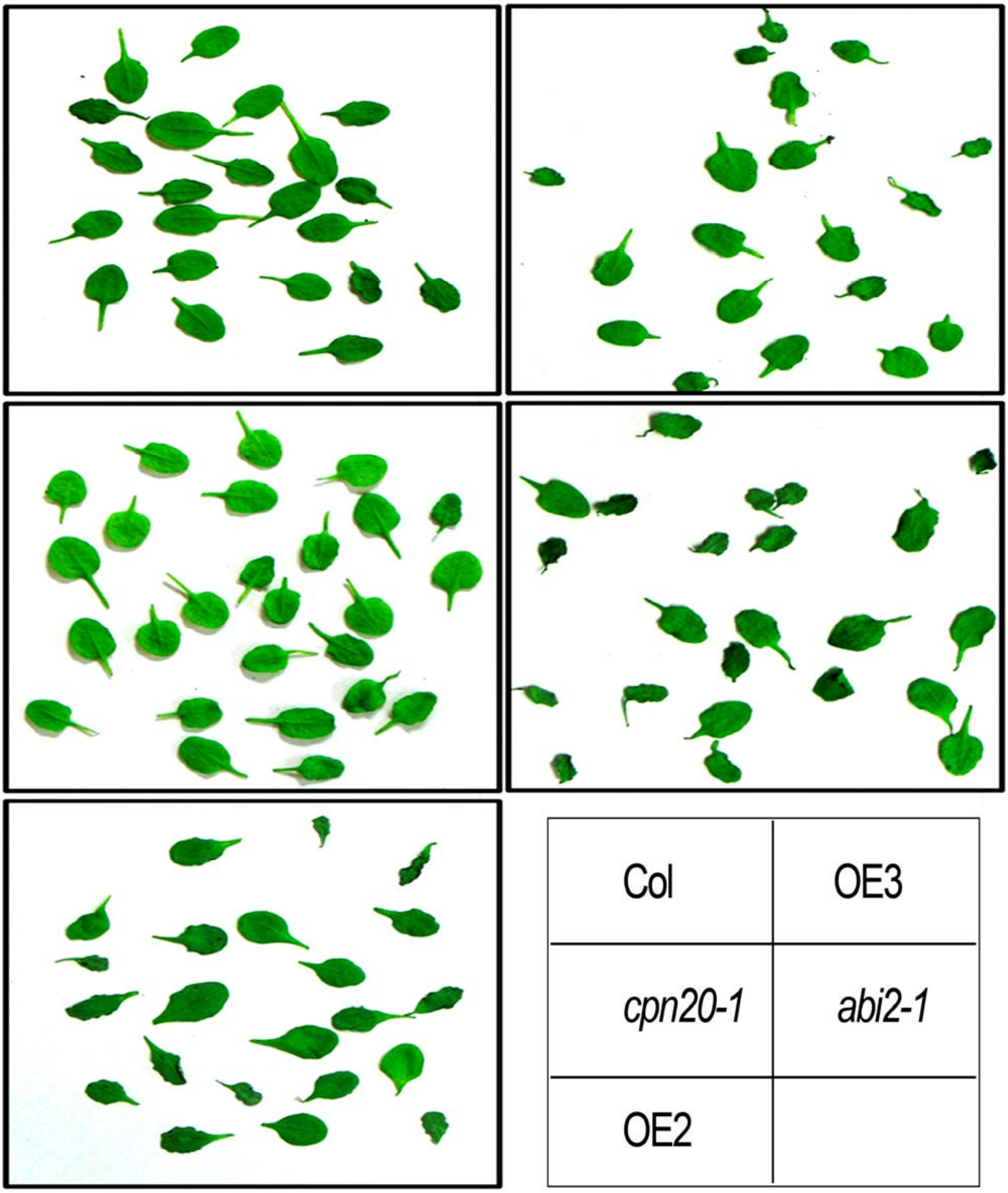

Supplementary Fig. 6. Status of the Detached Leaves of the Different Genotypes in a Water Loss Assay.
The plants of wild-type Col, *cpn20-1* mutant, two CPN20-overexpressing lines (OE2 and OE3) and *abi2-1* mutant were subjected to a 6-hour period water loss assay as described in **Fig. 2F**. The experiment was repeated five times with similar results.

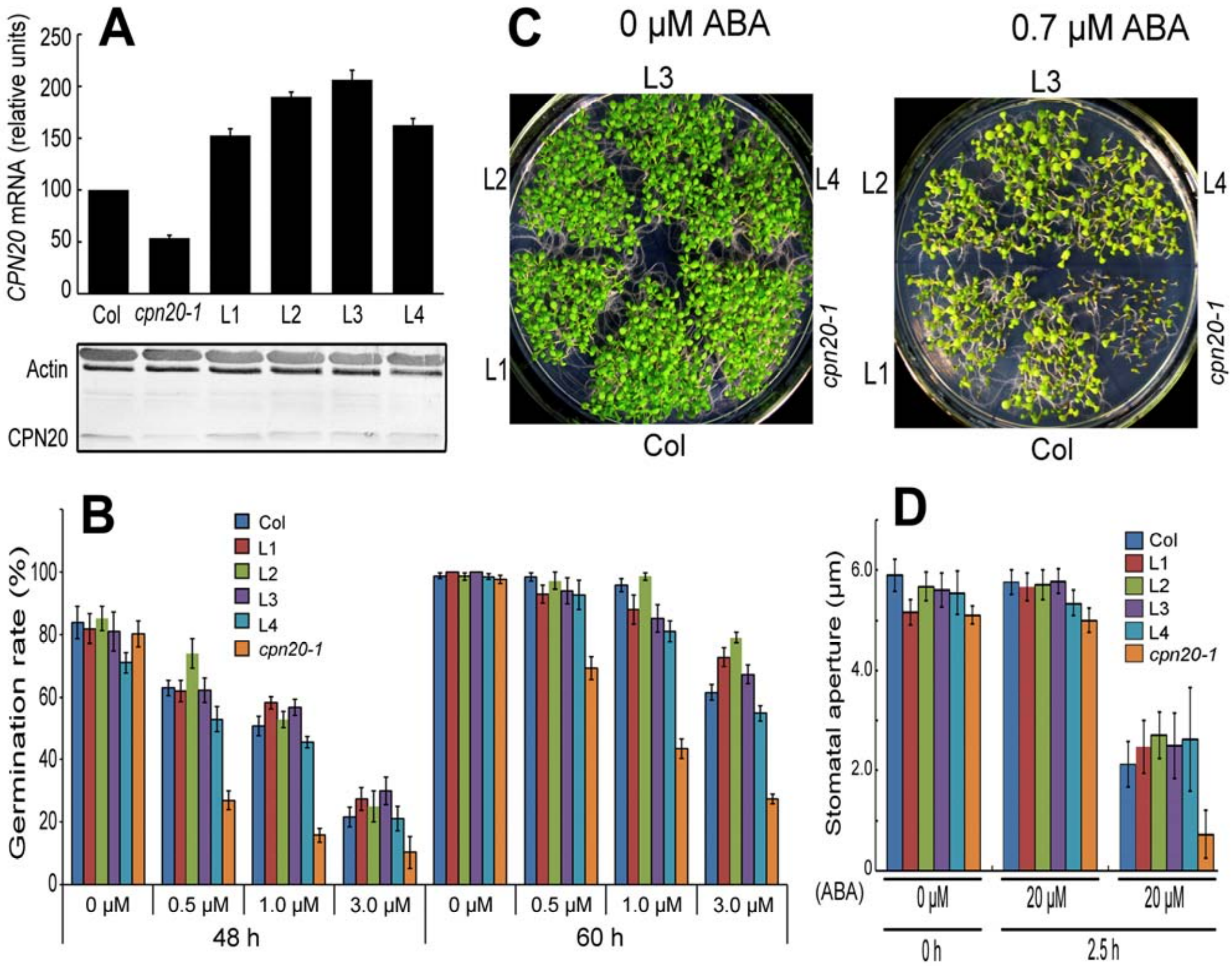

Supplementary Fig. 7. Phenotypic Analysis of the *cpn20-1* Complemented Lines.

(A) Quantitative real-time PCR (top) and immunoblotting (bottom) analysis for *CPN20* expression in the *cpn20-1* mutant. The value of *CPN20* mRNA obtained from the 4-week-old Col seedlings was taken as 100%, and all the other values were normalized relative to this value. Note that the expression levels of *CPN20* mRNA in the transgenic complemented lines (L1-L4) are higher than those of wild-type Col plants, but the CPN20 protein levels in the complemented lines are essentially similar to those of wild-type Col plants. Error bars represent SE of three independent biological determinations. **(B)** Seed germination rates of the wild-type Col, *cpn20-1* mutant and different complemented lines in MS medium supplemented with 0, 0.5, 1 or 3 μM (±)ABA from 48 and 60 h after stratification. Error bars represent SE of three independent biological determination. **(C)** Post-germination growth of the wild-type Col, *cpn20-1* mutant and different complemented lines in the ABA-free (0 μM ABA) and 0.7 μM-ABA-containing medium 12 days after stratification. **(D)** ABA-induced stomatal closure of the wild-type Col, *cpn20-1* mutant and different complemented lines. Values are the means ± SE of five independent experiments; n = 60 apertures per experiment.

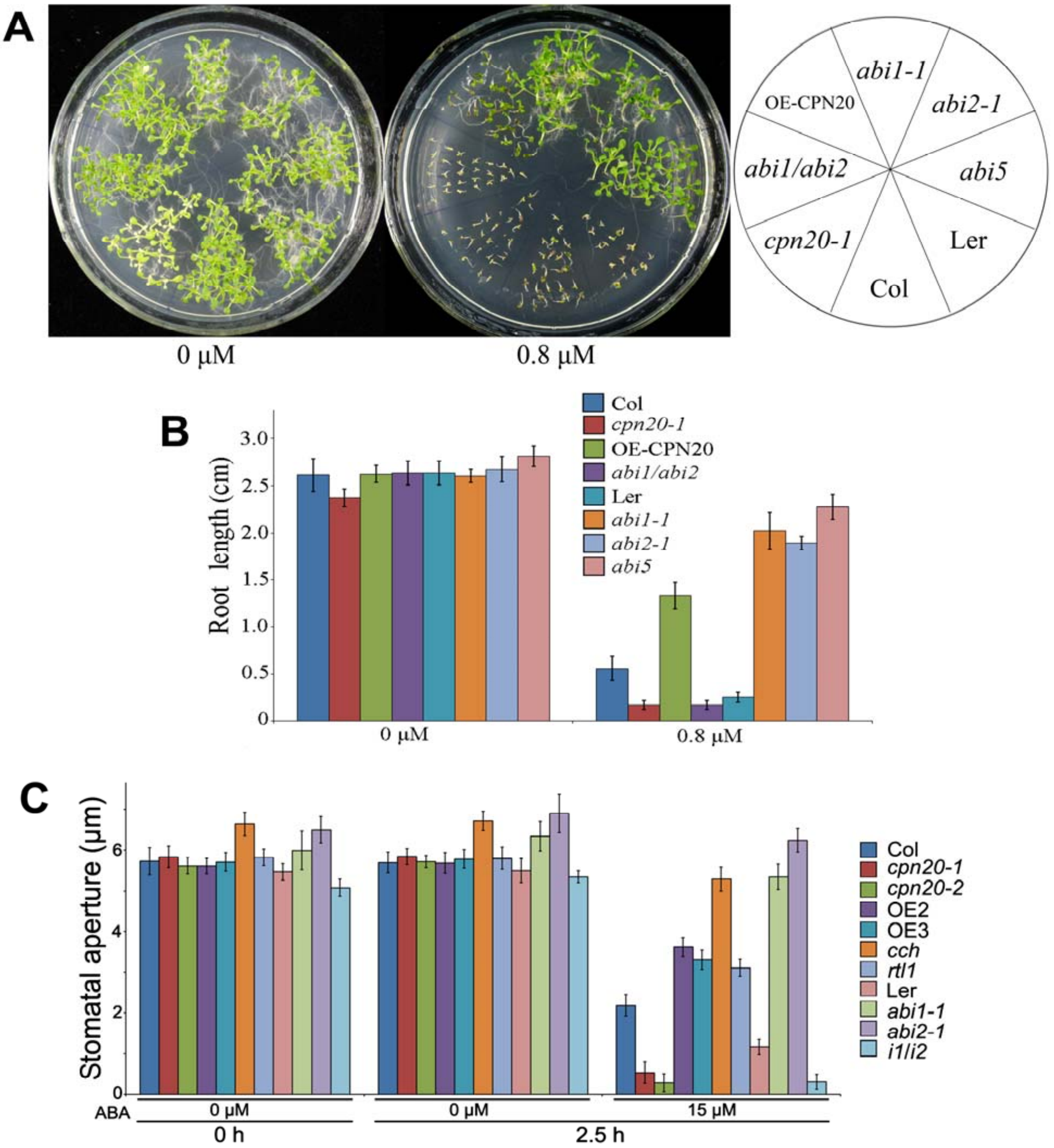

Supplementary Fig. 8. Comparison of ABA Responses among Different Genotypes.
(A) Post-germination growth of the wild-types Col and Ler, *cpn20-1, abi1-1*(the *abi1* dominant mutant)*, abi2-1*(the *abi2* dominant mutant)*, abi5* single mutants, *abi1 abi2* double mutant and a *CPN20*-overexpressing line (OE-CPN20) in the ABA-free (0 µM ABA) and 0.8 µM-ABA-containing medium 12 days after stratification. **(B)** Root length of the different genotypes described above in **(A)**. Values are the means ± SE of five independent experiments. **(C)** Stomatal aperture of the wild-type plants (Col and Ler ecotypes), *cpn20-1*, *cpn20-2*, two *CPN20*-overexpression lines (OE2 and OE3), *cch*, *rtl1*, *abi1-1*(the *abi1* dominant mutant), *abi2-1*(the *abi2* dominant mutant), *abi1 abi2* (*i1/i2*) double mutant. The *i1/i2* double mutant is generated by crossing from *abi1* (SALK_076309) and *abi2* (SALK_015166); both *abi* mutants are T-DNA insertional mutants, and the *abi1* is a knockout allele, and *abi2 is* a knockdown allele with a decrease of about 60% in *ABI2* mRNA. The *abi1-1* and *abi2-1* dominant mutants are from Ler background and other mutants from Col background. Values are the means ± SE of five independent experiments; n = 60 apertures per experiment.

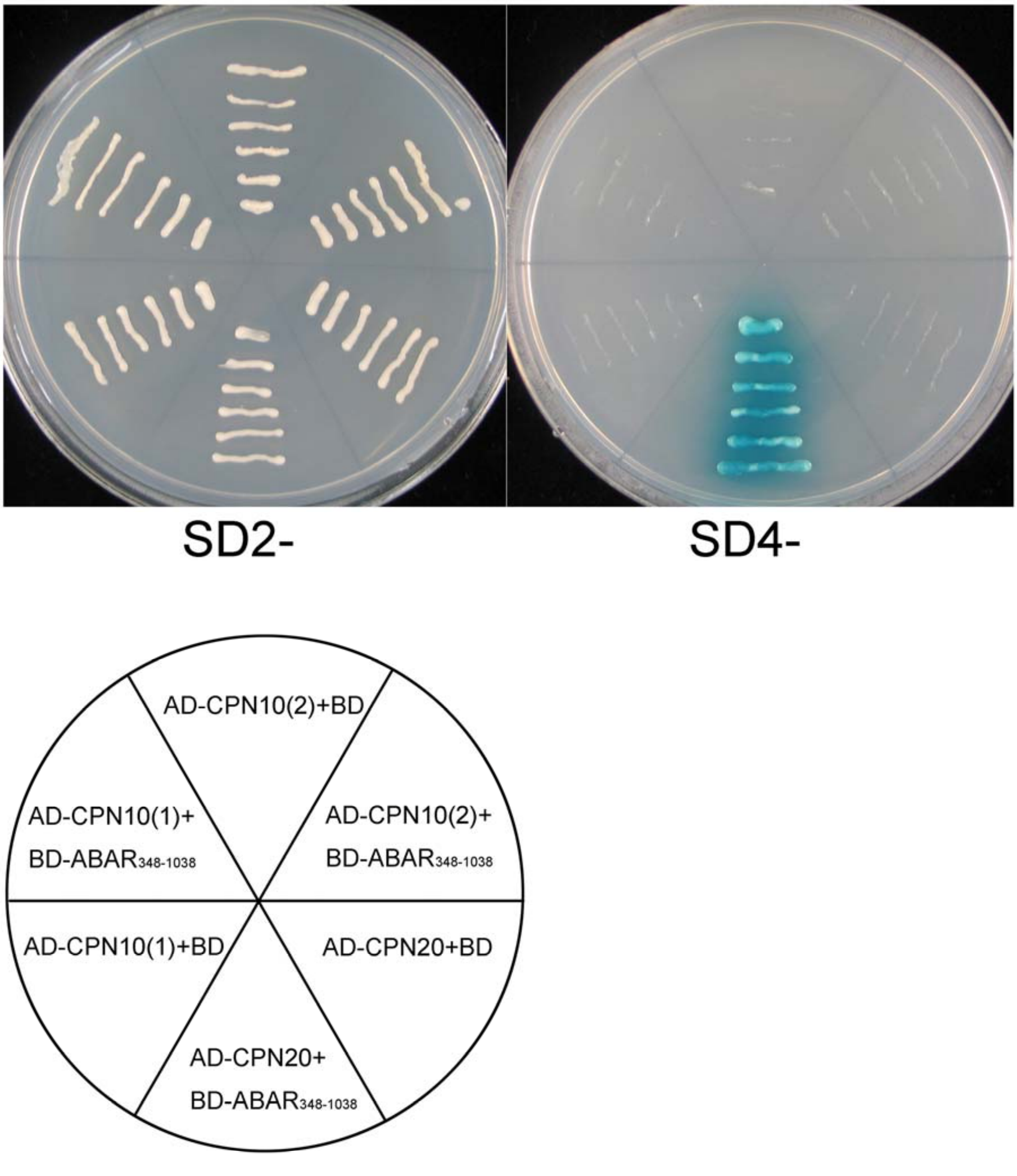

Supplementary Fig. 9. Neither CPN10(1) Nor CPN10(2) Interacts with ABAR.
The open reading frame of the middle fragment (amino acid residues 348-1038) of *ABAR* was fused with the DNA binding domain in the bait vector pGBKT7 (BD-ABAR$_{348\text{-}1038}$), while the full-length sequence of *CPN10(1)* or *CPN10(2)* was fused with activation domain in the prey vector pGADT7 [AD-CPN10(1) or AD-CPN10(2)]. The yeast strain AH109 co-transformed with the AD-CPN10(1) /BD-ABAR$_{348\text{-}1038}$ or AD-CPN10(2)/BD-ABAR$_{348\text{-}1038}$ construct pairs was able to grow on the medium lacking Trp and Leu (SD2-), but not on the medium lacking Trp, Leu, His, and Ade (SD4-). AD-CPN20/BD, AD-CPN10(1) /BD and AD-CPN10(2)/BD were taken as negative controls. AD-CPN20/BD-ABAR$_{348\text{-}1038}$ was taken as a positive control. The experiments were repeated three times with the same results.

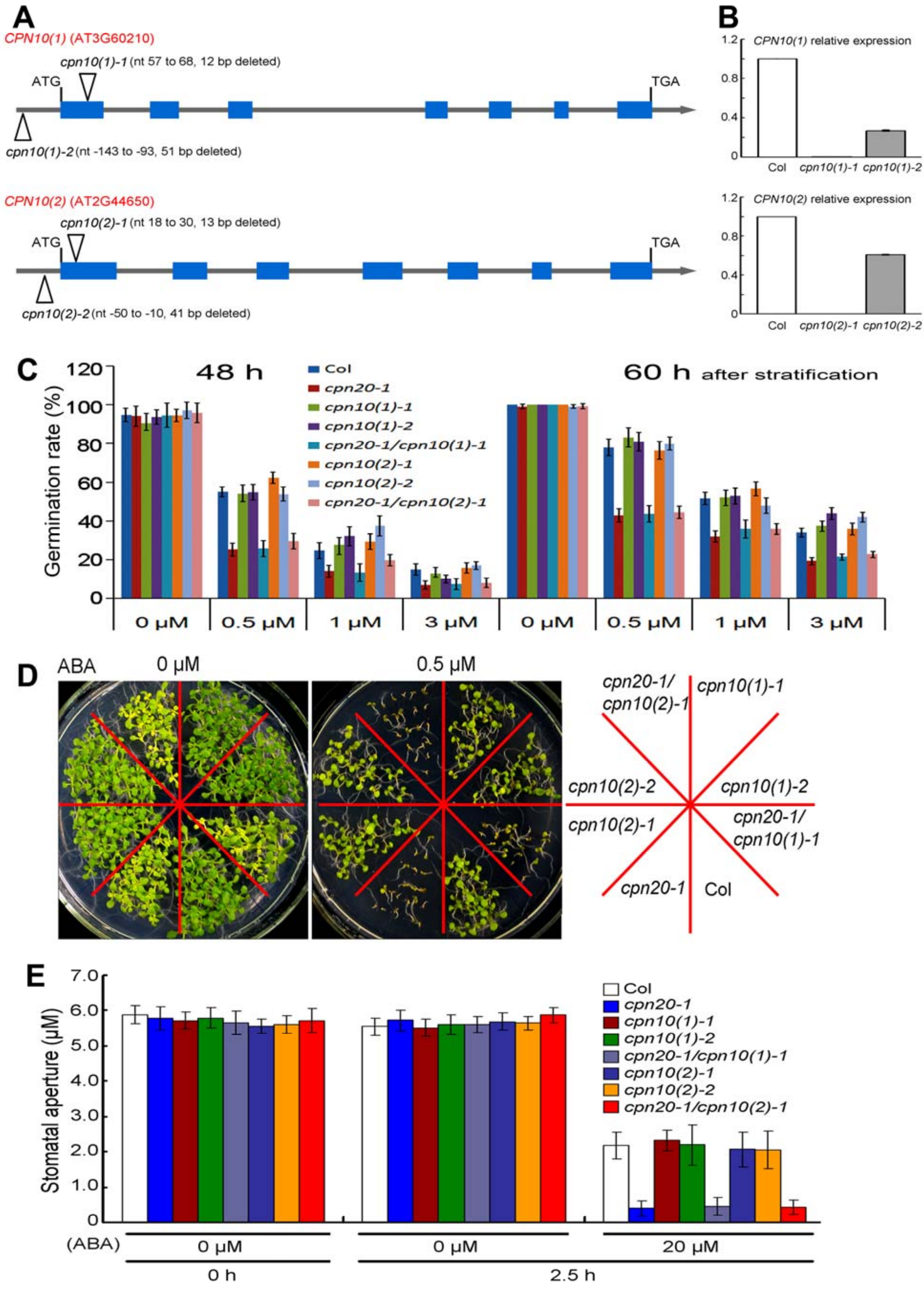

Supplementary Fig. 10. Downregulation or Disruption of CPN10s Does Not Alter ABA Sensitivity.

(A) T-DNA insertion sites in *cpn10(1)-1* (SALK_093761), *cpn10(1)-2* (SALK_027792C), *cpn10(2)-1* (SALK_023440) and *cpn10(2)-2* (SALK_051544). Blue boxes and grey lines represent exons and introns,

respectively. The T-DNA insertion sites are indicated by open arrowheads, and nt indicates nucleotides. In *cpn10(1)-1* mutant, the T-DNA was inserted into the first exon at nt 57 to 68 of *CPN10(1)* gene with a 12-bp fragment deleted. In *cpn10(1)-2* mutant, the T-DNA was inserted into the promoter region at nt -143 to -93 of the translation start codon (ATG) of *CPN10(1)* gene with a 51-bp fragment deleted. In *cpn10(2)-1* mutant, the T-DNA was inserted into the first exon at nt 18 to 30 of *CPN10(2)* gene with a 13-bp fragment deleted. In *cpn10(2)-2* mutant, the T-DNA was inserted into the promoter region at nt -50 to -10 upstream of the start codon ATG of *CPN10(2)* gene with a 41-bp fragment deleted. **(B)** Quantitative real-time PCR analysis for *CPN10(1)* and *CPN10(2)* expression in the *cpn10s* mutants. The value obtained from the 4-week-old Col seedlings was taken as 100%, and all the other values were normalized relative to this value. Note that *cpn10(1)-1* and *cpn10(2)-1* are knockout mutant alleles while *cpn10(1)-2* and *cpn10(2)-2* are knockdown alleles. Error bars represent SE of three independent biological determinations. **(C)** Seed germination rates of the wild-type Col and different mutants in MS medium supplemented with 0, 0.5, 1 or 3 μM (±)ABA from 48 and 60 h after stratification. Error bars represent SE of three independent biological determination. **(D)** Seed germination and post-germination growth of the wild-type plants (Col), *cpn10(1)-1*, *cpn10(1)-2*, *cpn10(2)-1* and *cpn10(2)-2* single mutants, and *cpn20-1 cpn10(1)-1* and *cpn20-1 cpn10(2)-1* double mutants in the ABA-free (0 μM ABA) and 0.5μM-ABA-containing medium 12 days after stratification. **(E)** ABA-induced stomatal closure of the different genotypes mentioned in **(C)**. Values are the means ± SE of five independent experiments; n = 60 apertures per experiment.

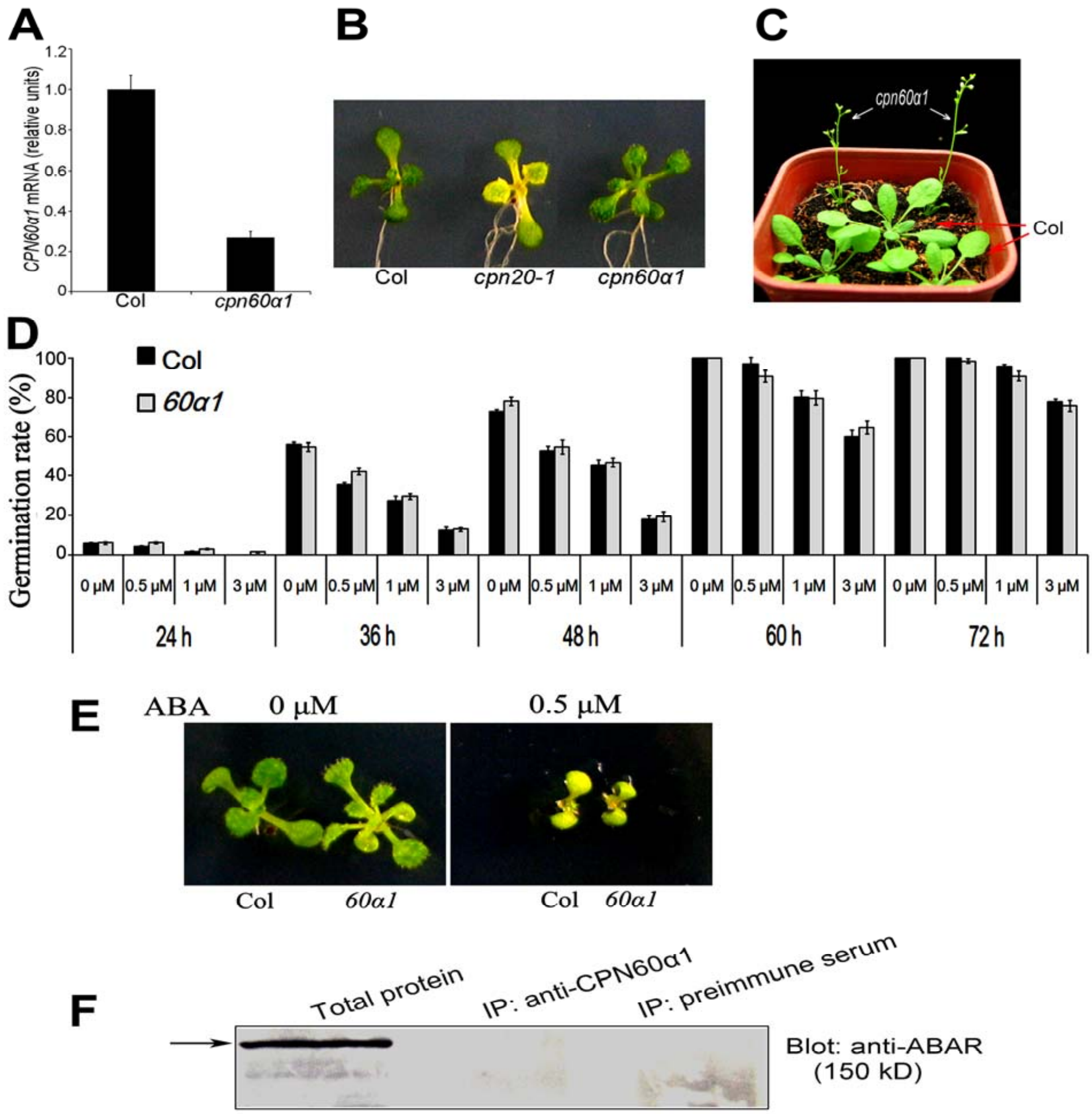

Supplementary Fig. 11. CPN60α1 Is Not Involved in ABA Signal Transduction.
(A) Expression of *CPN60α1* gene in Col and *cpn60α1* mutant (SALK_082308) determinate by quantitative real-time PCR. Note that the *cpn60α1* is a knockdown mutant allele. Each value is the mean ±SE of three independent biological determinations. **(B)** and **(C)** Comparison of the growth and developmental phenotypes of 10-day-old seedlings of the wild-type Col, *cpn20-1* and *cpn60α1* mutant **(B)** and 20-day old seedlings of the Col and *cpn60α1* mutant **(C)** in ABA-free medium. **(D)** Seed germination rates of the wild-type Col and *cpn60α1* mutant in MS medium supplemented with 0, 0.5, 1 or 3 μM (±)ABA from 24 to 72 h after stratification. Error bars represent SE of three independent biological determination. **(E)** Seedling growth of the wild-type Col and *cpn60α1* mutant in the ABA-free (0 μM ABA) and ABA-containing (0.5 μM ABA) medium 10 days after stratification. Seeds were directly planted in the ABA-free or ABA-containing medium, and seedling growth was investigated 10 days after stratification. **(F)** Co-immunoprecipitation (Co-IP) assays in plants: ABAR was not co-immuno-precipitated with CPN60α. The anti-CPN60α1 serum was used in the Co-IP assay in the total protein from the wild-type Col plants. Immuno-precipitates (IP: anti-CPN60α1) was immuno-blotted with anti-ABAR serum (Blot: anti-ABAR). IP with preimmune serum was taken as a negative control.

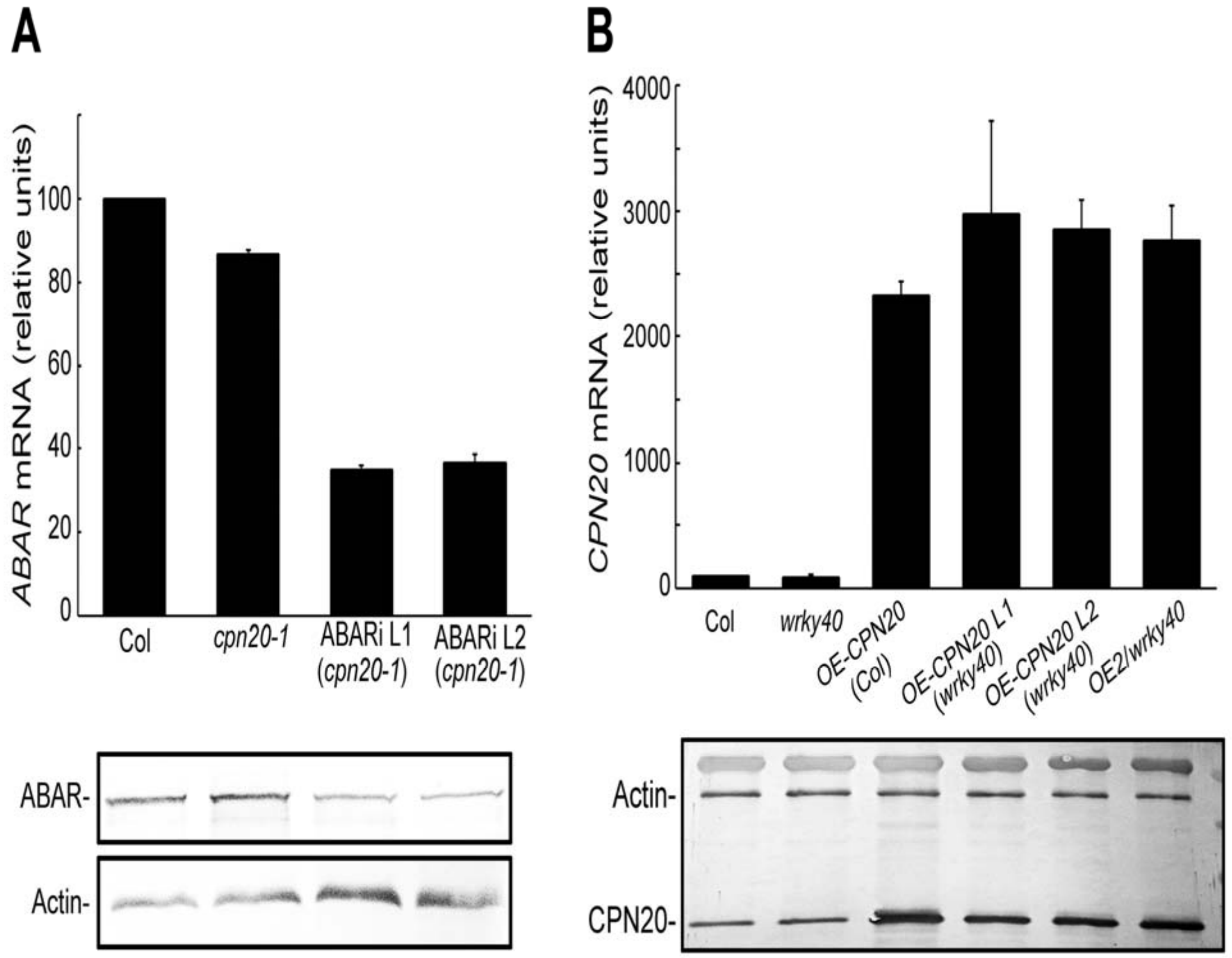

Supplementary Fig. 12. Real-time PCR and Immunoblotting Analysis of Different Mutants and Transgenic Lines. **(A)** Expression of *ABAR* gene in *cpn20-1* mutant and ABAR-RNAi lines in the *cpn20-1* mutant background [ABARi L1/L2 (*cpn20-1*)]. **(B)** Expression of *CPN20* gene in *wrky40* mutant, the *CPN20*-overexpression (OE-CPN20) line in the Col background [OE-CPN20 (Col)], the OE-CPN20 lines in the *wrky40* mutant background [OE-CPN20 L1/L2 (*wrky40*)] and OE-CPN20 *wrky40* double mutant (OE2/*wrky40*).

Top panels, real-time PCR data. Values are the means ± SE of five independent determinations. Bottom panels, immunoblotting data. The experiments were repeated three times with similar results.

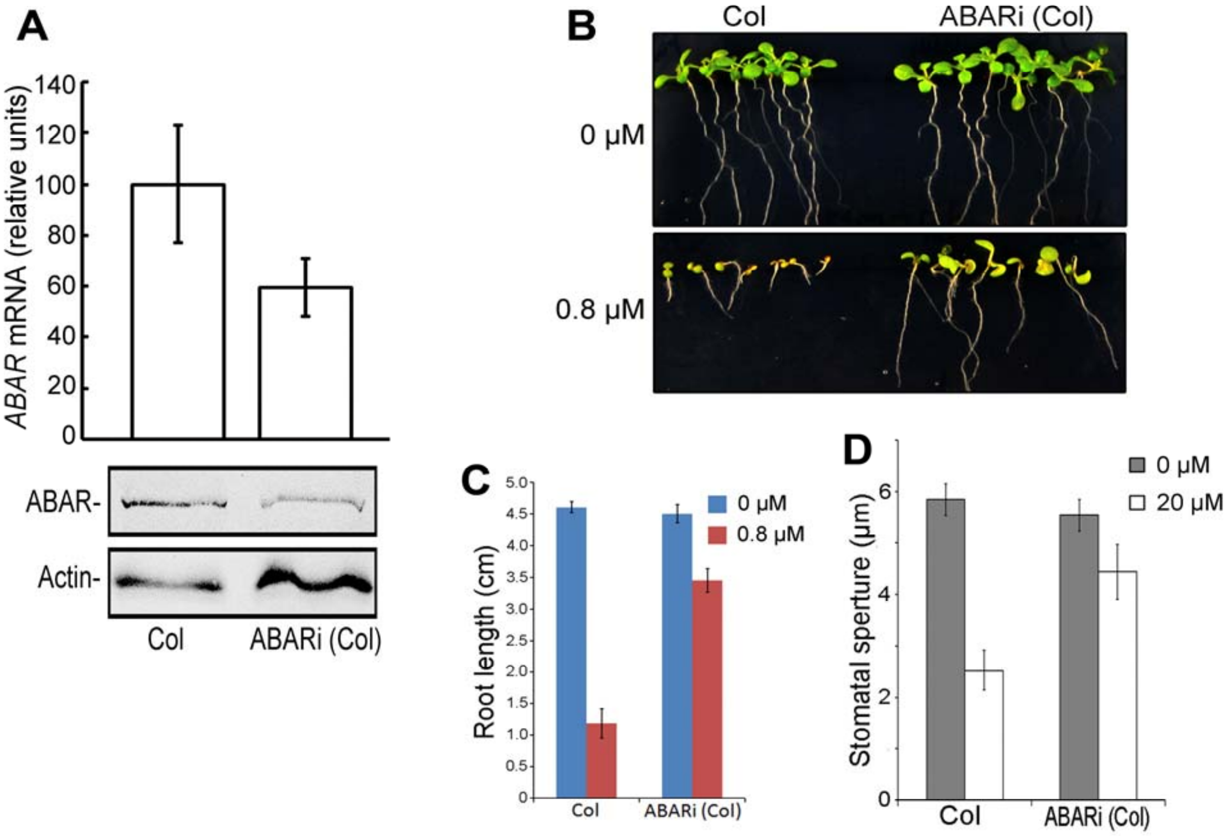

Supplementary Fig. 13. ABA-Related Phenotypes of the *ABAR*-RNAi Line Described in Fig. 4.
(A) Analysis of the *ABAR* expression of the *ABAR*-RNAi line under Col background. Top columns, *ABAR* mRNA levels, and bottom panel, ABAR protein levels in Col and the RNAi line. The Actin protein was used as a loading control. **(B)** Seed germination and early seedling growth of the wild-type Col and the *ABAR*-RNAi line [ABARi (Col)] in ABA-free (0 μM) and ABA-containing (0.8 μM) medium 10 days after stratification. **(C)** Statistics of the data described in **(B)**. Values are the means ± SE of five independent determinations. **(D)** ABA-induced stomatal closure of the wild-type Col and the *ABAR*-RNAi line. Values are the means ± SE of five independent experiments; n = 60 apertures per experiment.

Supplementary Table 1. Primers Used in This Study

1. Primers for Analysis of Protein Interaction by Yeast Two-Hybrid System

$ABAR_{348\text{-}1038}$	Forward primer:	5’-CCGGAATTCGAGCTTGAGGCTAG-3’
	Reverse primer:	5’-ACGCGTCGACGTTGTCAGTTCCCCAAAG-3’
CPN20	Forward primer:	5’-GGAATTCATGGCGGCGACTCA-3’
	Reverse primer:	5’-CCGCTCGAGCTAAGAAAGTATA-3’

2. Primers for Production of Anti-CPN20 Serum

Forward primer:	5’- CCGGAATTC ATGGCGGCGACTCAACT-3’
Reverse primer:	5’- CCGCTCGAGCTAAGAAAGTATAGCCATCACAT-3’

3. **Primers for Analysis of T-DNA Flanking Sequence**

LBa1:	5’-GGTTCACGTAGTGGGCCATC-3’
RBa1:	5’-GTTTCTGACGTATGTGCTTAGC-3’
LB1:	5'-GCCTTTTCAGAAATGGATAAATAGCCTTGCTTCC-3'
RB1:	5’-ATTAGGCACCCCAGGCTTTACACTTTATG-3’

4. **Primers for Analysis of the *CPN20* and *CPN10* Genomic Sequences**

LP1 (for *cpn20-1*):	5’- TTCCGACACGTTTGGTTCTAC-3’
RP1 (for *cpn20-1*):	5’- TGACATAGTCACTGGTGACGC-3’
LP1 (for *cpn20-2*):	5’- TGAGATGGTTTTTGTTCTGACC-3’
RP1 (for *cpn20-2*):	5’- ACAGAAGCAGCTTTGACAACC-3’
LP1 [for *cpn10 (1)-1*]:	5’- ACATGTCCAATTCCAAAGCTG-3’
RP1 [for *cpn10 (1)-1*]:	5’- AGAAAGCTAAGGAATCACCGG-3’
LP1 [for *cpn10 (1)-2*]:	5’-TAACCAAAACATTCGTGGCTC-3’
RP1 [for *cpn10 (1)-2*]:	5’- GACCCTGTCTGCTTGTGGTAC-3’
LP1 [for *cpn10 (2)-1*]:	5’-TACCCGATCAAGGTTCTGTTG-3’
RP1 [for *cpn10 (2)-1*]:	5’-AAGAGCCCATAAGTTTTTGGC-3’
LP1 [for *cpn10 (2)-2*]:	5’-TACCCGATCAAGGTTCTGTTG-3’
RP1 [for cpn10 (2)-2]:	5’AAGAGCCCATAAGTTTTTGGC-3’

5. **Primers for Generation of *CPN20*-overexpressing Transgenic Plants**

Forward primer:	5’- GGACTAGTATGGCGGCGACTCAACT-3’
Reverse primer:	5’- CGGGGTACCGGAGAAAGTATAGCCATCACATCTG-3’

6. **Primers for the Test of Protein-Protein Interaction by LCI**

ABAR-NLuc:	Forward primer:	5’-GGGGTACCATGGCTTCGCTTGTGT-3’
	Reverse primer:	5’-ACGCGTCGACTCGATCGATCCCTTC-3’
CLuc-CPN20	Forward primer:	5’-CGGGGTACC ATGGCGGCGACTCAACT-3’
	Reverse primer:	5’-GCGTCGACCTAAGAAAGTATAGCCATCACAT-3’

7. **Primers for *CPN20*-promoter-GUS Staining Analysis**

Forward primer:	5’- ACGCGTCGACAAAATTGGCCGTGGAATTGG-3’
Reverse primer:	5’- CCGGAATTCTTCTCCTATCCGTCAAAACAC-3’

8. **Primers for Transient Expression in *Arabidopsis* Protoplasts**

CPN20-GFP	Forward primer:	5’- TGCTCTAGAATGGCGGCGACTCAACT-3’
	Reverse primer:	5’- CGGGGTACCAGAAAGTATAGCCATCACATCTG-3’
CPN20-mCherry	Forward primer:	5’-CCTTAATTAAATGGCGGCGACTCAACT-3’
	Reverse primer:	5’- TTGGCGCGCCTAGAAAGTATAGCCATCACATCTG-3’

9. **Gene-Specific Primers for Real-Time PCR Analysis**

ACTIN2/8	Forward primer:	5’- GGTAACATTGTGCTCAGTGGTGG-3’
	Reverse primer:	5’- AACGACCTTAATCTTCATGCTGC-3’
CPN20 (At5g20720)	Forward primer:	5’- ATGGCGGCGACTCAACTTACAGCG-3’
	Reverse primer:	5’- GACAACCAAACGACGGAACTGGCTC-3’
ABF1 (At1g49720)	Forward primer:	5’- TCAACAACTTAGGCGGCGATAC-3’
	Reverse primer:	5’- GCAACCGAAGATGTAGTAGTCA-3’
ABF2 (At1g45249)	Forward primer:	5’- TTGGGGAATGAGCCACCAGGAG-3’
	Reverse primer:	5’- GACCCAAAATCTTTCCCTACAC-3’
ABF3 (At4g34000)	Forward primer:	5’- CTTTGTTGATGGTGTGAGTGAG-3’
	Reverse primer:	5’- GTGTTTCCACTATTACCATTGC-3’
ABF4 (At3g19290)	Forward primer:	5’- AACAACTTAGGAGGTGGTGGTC-3’
	Reverse primer:	5’- CTTCAGGAGTTCATCCATGTTC-3’
ABI4 (At2g40220)	Forward primer:	5’- GGGCAGGAACAAGGAGGAAGTG-3’
	Reverse primer:	5’- ACGGCGGTGGATGAGTTATTGAT-3’
ABI5 (At2g36270)	Forward primer:	5’- CAATAAGAGAGGGATAGCGAACGAG-3’
	Reverse primer:	5’- CGTCCATTGCTGTCTCCTCCA-3’
MYB2 (At2g47190)	Forward primer:	5’- TGCTCGTTGGAACCACATCG-3’
	Reverse primer:	5’- ACCACCTATTGCCCCAAAGAGA-3’
RD29A (At5g52310)	Forward primer:	5’- ATCACTTGGCTCCACTGTTGTTC-3’
	Reverse primer:	5’- ACAAAACACACATAAACATCCAAAGT-3’
SnRK2.2 (At3g50500)	Forward primer:	5’- ATATGCCATCGGGATCTGAA-3’
	Reverse primer:	5’- TTGGTTGGGAATGAAGAACAG-3’
SnRK2.3 (At5g66880)	Forward primer:	5’- GTTGGATGGAAGTCCTGCTC-3’
	Reverse primer:	5’- TGCCATCATATTCCTGACGA-3’
SnRK2.6 (At4g33950)	Forward primer:	5’- TGGAGTTGCGAGATTGATGAGAG-3’
	Reverse primer:	5’- CCTGTGGTTGATTATCTCCCTTTTT-3’
